# Blood N-glycomics reveals individuals at risk for cognitive decline and Alzheimer’s disease

**DOI:** 10.1016/j.ebiom.2025.105598

**Published:** 2025-02-20

**Authors:** Robin Ziyue Zhou, Stefan Gaunitz, Bjørn-Eivind Kirsebom, Britt Lundin, Marie Hellström, Alenka Jejcic, Anders Sköldunger, Anders Wimo, Bengt Winblad, Tormod Fladby, Sophia Schedin-Weiss, Lars O. Tjernberg

**Affiliations:** a Division of Neurogeriatrics, Department of Neurobiology, Care Sciences, and Society, Karolinska Institutet, Solna, Sweden; b Clinical Chemistry, Medical Diagnostics Karolinska, Karolinska University Laboratory, Karolinska University Hospital, Stockholm, Sweden; c Division of Clinical Chemistry, Department of Laboratory Medicine, Karolinska Institutet, Stockholm, Sweden; d Department of Neurology, University Hospital of North Norway, Tromsø, Norway; e Department of Psychology, Faculty of Health Sciences, The Arctic University of Norway, Tromsø, Norway; f Department of Neurology, Akershus University Hospital, Lørenskog, Norway; g Institute of Clinical Medicine, University of Oslo, Oslo, Norway; h Theme Inflammation and Aging, Karolinska University Hospital, Huddinge, Sweden

**Keywords:** Alzheimer's disease, Biomarkers, Cognitive decline, Dementia, Glycomics, N-glycosylation

## Abstract

**Background:**

Blood biomarkers with prognostic accuracy for Alzheimer's disease (AD) are crucial for selecting at-risk individuals for interventions. Altered protein N-glycosylation has been implicated in several pathogenic pathways in AD and could be an early AD biomarker.

**Methods:**

We developed a mass spectrometry-based method to simultaneously quantify 62 blood N-glycan structures in individuals with biological or clinical AD and matched controls. We analysed N-glycan levels in a Swedish discovery cohort (n = 40) and validated our results in a Norwegian cohort (n = 60). Individuals were grouped according to N-glycan levels using unsupervised hierarchical clustering. Difference in disease progression between groups were modelled using linear mixed-effects models.

**Findings:**

A subgroup of individuals exhibited low blood N-glycosylation (32.4% of Swedish cohort, 37.9% of Norwegian cohort). In the Swedish cohort, low N-glycosylation was associated with AD and cognitive decline. In the Norwegian cohort, low blood N-glycosylation showed no correlation with amyloid/tau, but importantly, strongly predicted future cognitive decline. In total, fourteen N-glycan structures were significantly less abundant in the low N-glycosylation group compared to the rest of the individuals in both cohorts.

**Interpretation:**

Reduced blood N-glycan levels predict cognitive decline independent of amyloid or tau status. Blood N-glycome profiling could be used to identify individuals at risk for AD dementia.

**Funding:**

Stiftelsen för Gamla Tjänarinnor, Stockholm County Council-ALF, JPND, PMI-AD, Medical Diagnostics Karolinska, Helse-Nord, Gun och Bertil Stohnes stiftelse, Demensförbundet, Stiftelsen Dementia, Margaretha af Ugglas’ foundation, Vinnova, the private initiative “Innovative ways to fight Alzheimer's disease–Leif Lundblad Family and others”.

**Research in context**

**Evidence before this study**

It is increasingly clear that aberrant N-glycosylation is present on key proteins in Alzheimer's disease (AD) and may be an early triggering event of AD pathogenesis. However, few studies have explored the utility of N-glycan assays in detecting AD. A PubMed search conducted on October 3, 2024 with the terms (“Alzheimer” OR “Alzheimer's”) AND “N-glycosylation” revealed 67 results. Previous studies on the N-glycome of AD patients have shown altered N-glycosylation in brain and cerebrospinal fluid. Further studies have reported changes in the glycosylation of specific proteins in blood in AD. Although some studies have reported on levels of blood N-glycan structures as possible biomarkers for AD, none of these studies have validated their findings in multiple cohorts. Thus, a comprehensive investigation of the blood N-glycome within the context of AD is missing.

**Added value of this study**

We developed a state-of-the-art N-glycan quantification method based on porous graphitic carbon liquid chromatography coupled to mass spectrometry (PGC-LC-MS). Using this method, we quantified the levels of 62 unique N-glycan structures in blood of AD patients and controls in two separate cohorts. Using tandem mass spectrometry, we estimated the structures of these 62 N-glycans. Decreased levels of a group of 35 N-glycans were associated with clinical AD dementia and lower cognitive function in an older Swedish cohort, while lower levels of 22 N-glycans were associated with *APOE4* genotype and cognitive decline in a younger Norwegian validation cohort. In summary, low blood N-glycosylation was associated with poor prognosis in AD, and 14 N-glycan structures were decreased in the low N-glycosylation group of both cohorts.

**Implications of all the available evidence**

Our study provides the most extensive characterization of the blood N-glycome in AD to date. We show that low blood N-glycosylation is a prognostic marker for future cognitive decline, providing additional information aside from amyloid/tau status. Blood N-glycome profiling could be developed as a less-invasive and easily accessible prognostic biomarker in AD, thus aiding in precision medicine approaches. Furthermore, our method could be utilized as a useful reference for the development of glycan biomarkers for AD and other diseases. Finally, our findings confirm that alterations in N-glycosylation can be found early in the disease course of AD, thus opening new avenues to explore regarding glycosylation-targeting therapies against AD.

## Introduction

Alzheimer's disease (AD) is a neurodegenerative disease that accounts for more than half of dementia cases worldwide.^1^ The disease is characterised by extracellular plaques in brain consisting of amyloid β-peptide (Aβ) and intraneuronal neurofibrillary tangles (NFTs) containing hyperphosphorylated tau.^2^ Recently, clinical trials showed that monoclonal antibodies directed against Aβ slows disease progression in AD.^3^, ^4^, ^5^ However, some subgroups, most notably *APOE4* carriers, exhibit lower treatment response and higher frequency of amyloid-related imaging abnormalities (ARIA).^6^^,^^7^ Cerebrospinal fluid (CSF) proteomics studies have revealed distinct molecular subtypes in AD,^8^^,^^9^ further emphasising the need to develop biomarkers to risk-stratify individuals and select responsive populations for treatment.

Glycans are post-translational modifications on proteins and have important roles for protein trafficking and function.^10^^,^^11^ In the context of AD, glycosylation affects the metabolism and function of several proteins implicated in AD pathogenesis, including beta-site amyloid precursor protein cleaving enzyme 1 (BACE1),^12^ tau,^13^, ^14^, ^15^ nicastrin,^16^ triggering receptor expressed on myeloid cells 2 (TREM2),^17^ and the amyloid precursor protein (APP).^18^ Changes in the N-glycome have been detected in AD brain^19^, ^20^, ^21^, ^22^ and N-glycosylation in cerebrospinal fluid (CSF) has been studied as a potential biomarker for AD.^23^ We and others have previously reported that the bisecting N-acetylglucosamine (GlcNAc) N-glycan epitope is elevated in CSF in AD,^24^, ^25^, ^26^ and that elevated bisecting GlcNAc levels could predict cognitive decline already at an amyloid/tau negative stage.^27^

Recent advances in blood-based biomarkers allow classification of individuals according to amyloid/tau (A/T) pathology, neurodegeneration (N), and microglial activation, thus enabling characterisation of AD-related pathology based on blood tests.^28^, ^29^, ^30^ The blood N-glycome or glycoproteome may provide additional information on AD subtype and pathological processes. The blood N-glycome is altered in cancer,^31^ and several glycoproteins are established markers for cancer.^32^^,^^33^ In the context of AD, altered levels of N-glycan structures or N-glycopeptides have been observed in blood.^34^, ^35^, ^36^, ^37^, ^38^ However, N-glycomic or N-glycoproteomic studies in AD have had small sample sizes, reported glycan composition rather than glycan structure, and/or lacked other available AD biomarkers to characterise study participants.

In the present study, we aimed to provide the most extensive blood N-glycomics investigation to date in AD. To achieve this goal, we developed a liquid chromatography-mass spectrometry (LC-MS) method using porous graphitic carbon (PGC) based chromatography, which enabled us to simultaneously quantify 62 distinct N-glycan structures in blood. Using this method, we analysed blood samples from a discovery cohort of 40 Swedish community-dwelling individuals characterised as clinical AD or controls. Moreover, we validated the findings in an AD biomarker-characterised Norwegian cohort consisting of 60 individuals classified as amyloid (A) or tau (T) positive or negative (A-T-, A+T-, or A+T+).

## Methods

### Study participants

In this study, samples from two separate cohorts were selected for analysis. Demographic data on the two cohorts are summarised in Table 1. To evaluate blood N-glycosylation patterns in patients with AD dementia, we analysed 40 plasma samples from community-dwelling individuals in Nordanstig, Sweden from the Swedish National study on Aging and Care in Nordanstig (SNAC-N) cohort.^39^ Participants with AD and controls were matched by age and sex, since these are known confounding factors when assessing serum N-glycan expression.^40^ To validate our findings in the SNAC-N cohort, we also selected 60 serum samples from the Norwegian Dementia Disease Initiation (DDI) study,^41^ conducted at memory clinics in Norway. Participants in the DDI study were characterised with AD biomarkers. We selected 20 individuals each in the A-T-, A+T-, and A+T+ groups, who were matched by age, sex, and years of education. In both cohorts, selection of matched samples was performed manually, based on exact matches in age and sex. If exact matches were not available, individuals were matched by nearest-neighbour matching. The study size was based on the availability of age- and sex-matched individuals in both cohorts. No pre-analytical power analysis was performed, as this was an exploratory study. To decrease the risk of Type I error, we instead sought to validate our findings in the Swedish SNAC-N cohort by reproducing the results in the Norwegian DDI cohort. Sample identity was blinded to the investigators during data collection.

**Table 1:** Cohort characteristics.

	Swedish discovery cohort SNAC-N (n = 40)		p-value	Norwegian validation cohort DDI (n = 60)			p-value
	Control (n = 20)	AD (n = 20)		A−T− (n = 20)	A+T− (n = 20)	A+T+ (n = 20)	
**Age (years)**							
Mean (SD)	81.6 (6.0)	82.8 (6.2)	0.47^a^	67.1 (7.5)	67.0 (8.2)	67.1 (6.8)	1.0^b^
Min, Max	72, 93	72, 90		51, 77	52, 79	52, 78	
Median (IQR)	81 (78–84)	82.5 (78.8–89.3)		67.5 (60.8–73.8)	68.5 (62.5–72.8)	66.5 (61.5–74.8)	
**Sex**							
Female (%)	13 (65%)	14 (70%)	0.74^c^	15 (75%)	15 (75%)	15 (75%)	1.0^d^
Male (%)	7 (35%)	6 (30%)		5 (25%)	5 (25%)	5 (25%)	
**Education (years)**							
Mean (SD)	8.1 (3.6)	6.2 (1.3)	0.16^a^	13.4 (2.3)	14.0 (3.2)	14.0 (2.2)	0.73^b^
Min, Max	5, 18	1, 8		10, 19	9, 20	10, 18	
Median (IQR)	6.5 (6–9)	6 (6–7)		13 (12–15)	13 (11.3–17)	14 (13–15)	
**Baseline MMSE**							
Mean (SD)	29.6 (0.6)	11.4 (6.0)	<0.0001^a^	29.2 (1.2)	27.8 (2.3)	27.1 (2.4)	0.0035^e^
Min, Max	28, 30	1, 19		26, 30	21, 30	21, 30	
Median (IQR)	30 (29–30)	13.5 (4.3–15.8)		30 (28–30)	28.5 (26.3–29)	27 (26–29)	
**APOE4 carriership**							
Frequency (%)	NA	NA	NA	9 (45%)	14 (70%)	16 (80%)	0.057^c^
**CSF Aβ42/Aβ40**							
Mean (SD)	NA	NA	NA	0.103 (0.009)	0.058 (0.013)	0.051 (0.010)	<0.0001^e^
Min, Max				0.09, 0.12	0.04, 0.08	0.04, 0.08	
Median (IQR)				0.10 (0.10–0.11)	0.05 (0.05–0.07)	0.05 (0.05–0.05)	
**CSF pTau181**							
Mean (SD)	NA	NA	NA	50.1 (10.5)	48.8 (9.2)	91.1 (17.7)	<0.0001^e^
Min, Max				17, 63	21, 62	70, 134	
Median (IQR)				52.5 (48–55.5)	50.5 (47–53.8)	88.5 (74.3–101.8)	
**CSF total tau**							
Mean (SD)	NA	NA	NA	300.0 (74.4)	302.6 (54.8)	663.3 (179.4)	<0.0001^e^
Min, Max				80, 394	129, 370	380, 1000	
Median (IQR)				305 (266.5–358.5)	315 (280–340.8)	640 (482.5–803.5)	

NA, not available.

a Mann-Whitney U-test.

b One-way ANOVA.

c Chi-squared test.

d Fisher's exact test.

e Kruskal-Wallis H-test.

### Ethics

The SNAC-N study received institutional review board (IRB) approval from the Swedish Central Ethical Review Board (IRB reference: Dnr Ö 26-2007). The DDI study received approval from the Regional Committees for Medical and Health Research Ethics in Norway (IRB reference: REK 2013/150). This ethical approval included recruitment of healthy controls, and the study adhered to the guidelines of the Norwegian Health and Research Act. Both studies were conducted in accordance with the Declaration of Helsinki, and all participants provided written informed consent prior to their involvement in the study. For glycan analysis in blood, IRB approval was obtained from Ethical Review Board in Stockholm, Sweden (IRB reference: 2013/1301-31/2).

### Discovery cohort: Swedish SNAC-N cohort

Swedish National Study on Aging and Care consists of four participating sites where SNAC-N is situated in the rural municipality of Nordanstig. It is formed by a panel of elders in different age cohorts (60, 66, 72, 78, 81, 84, 87, 90+) recruited between 2007 and 2009. Participants were randomly selected and followed up every three years for age cohorts 81+ and every six years for younger individuals. Data were collected to follow the ageing process from different aspects: health status, functional and cognitive ability, personality, social and economic situation, life habits, life satisfaction and perceived quality of life, use of drugs, and the formal and informal care and services received. All participants met a primary care physician who has a clinical and research background in dementia (AW). Data on cognitive function (e.g., Mini-Mental State Examination (MMSE), Clinical Dementia Rating—Sum of Boxes (CDR-SB)) were gathered in interviews by a trained nurse. Diagnosis of dementia was set with a protocol following the Diagnostic and Statistical Manual of Mental Disorders-IV (DSM-IV) criteria.^42^ For the diagnosis of probable or possible AD, the National Institute of Neurological and Communicative Disorders and Stroke and the Alzheimer's Disease and Related Disorders Association (NINCDS-ADRDA) criteria were applied.^43^ For staging, an algorithm based on the CDR-SB was used.^44^ The physician who set the diagnoses (AW) was blinded regarding the results of N-glycan analysis. Plasma samples were collected by an assistant nurse in ethylenediaminetetraacetic acid (EDTA) tubes and primarily frozen to −20 °C, followed by storage at −70 °C. Plasma phosphorylated tau 217 (pTau 217) was analysed in the Lumipulse fully automated platform G600II (Fujirebio Europe, Ghent, Belgium). Commercially available kits were used: Lumipulse G pTau 217 Plasma Immunoreaction Cartridges (Catalogue number 81472); Lumipulse pTau 217 Plasma Controls (Product number 81473) and Lumipulse G pTau 217 Plasma Calibrators (Product number 81471). The glial fibrillary acidic protein (GFAP) levels in plasma were quantified using the S-PLEX Neurology Panel 1 (human) Kit (MSD, MD, USA, Catalogue number K151ANV-1) according to manufacturer's manual and detected in the Meso QuickPlex SQ120 plate reader (MSD, MD, USA). The samples were diluted 1:2 in the buffer Diluent 100 provided in the kit. The data was subsequently analysed using the software MSD Discovery Workbench version 4.0 (MSD, MD, USA).

### Validation cohort: Norwegian DDI cohort

The Norwegian multi-centre DDI cohort consists of participants without dementia aged 40–80 years, with native languages of Norwegian, Danish, or Swedish. Recruitment occurred between 2013 and 2021 across six major university hospitals in Norway, drawing participants from memory clinics and local media advertisements. The cohort includes individuals with Subjective Cognitive Decline (SCD) or Mild Cognitive Impairment (MCI), classified according to established criteria, as well as healthy controls. Healthy controls were recruited from the spouses of patients, with additional participants sourced through local advertisements and orthopaedic patients who had lumbar punctures during surgery. MCI was determined when cognitive test results were 1.5 standard deviations below the normative mean within one or more cognitive domains, including delayed memory recall (Consortium to Establish a Registry for Alzheimer's disease (CERAD) word list test),^45^ executive function (Trail Making Test part B (TMT-B)),^46^ language/verbal fluency (Controlled Oral Word Association Test (COWAT)),^47^ and visuoperceptual ability (Visual Object and Space Perception Battery (VOSP) silhouettes).^48^ The cognitive screening battery in DDI also included MMSE and the clock test.^49^ Participants in DDI were followed up biennially with identical protocols, including neuropsychological tests and lumbar punctures.^41^ For the individuals included in the present study, cognitive status was followed for an average of 3.41 ± 2.3 years. Lumbar punctures were conducted between 9:00 AM and 12:00 PM, with CSF samples collected in sterile polypropylene tubes and then centrifuged. Samples were stored at −80 °C before analysis. Aβ42 and Aβ40 levels were measured using the QuickPlex SQ120 system from MesoScale Discovery (MSD, MD, USA) in a multiplex format, utilising the V-PLEX Aβ Peptide Panel 1 (6E10) kit (Catalogue number K15200E-1). All analyses followed the manufacturers' protocols. CSF concentrations of total tau (t-tau) and phosphorylated tau 181 (pTau 181) were determined using commercial enzyme-linked immunosorbent assays (ELISAs) from Innotest (Fujirebio, Ghent, Belgium), with t-tau measured via hTau Ag kits (Catalogue number 81572) and pTau 181 via 181P kits (Catalogue number 81574). An Aβ42/40 ratio cut-off of ≤0.077 was applied, as established in a previous [18F]-Flutemetamol PET study from the DDI cohort.^50^ Cut-off values for CSF t-tau (>378 ng/L) and pTau 181 (>66.5 ng/L) were determined based on receiver operating characteristic (ROC) analyses, comparing Aβ-negative healthy controls with Aβ-positive individuals with MCI or dementia within the DDI cohort.^51^ Serum samples were collected in Vacuette Serum Gel tubes (Catalogue number G454067, Greiner BioOne) and centrifuged for 10–15 min at room temperature. Collected serum samples were then aliquoted in Matrix tri-coded tubes (Catalogue number 479-1035, VWR) and stored at −80 °C before analysis. *APOE* genotyping was performed on EDTA blood samples as previously described.^41^

### Sample preparation

To release N-glycans from proteins, 10 μl of plasma or serum were added to a mixture of 164 μl of 0,1% RapiGest SF Surfactant (Waters, Catalogue number 186001861) in 40 mM Tris buffer pH 7.4, 20 μl of 100 mM dithiothreitol (Sigma Aldrich, Catalogue number 43816-50 ML), 5 μl of 50 ng/μl stachyose internal standard (Sigma Aldrich, Catalogue number S4001-100 MG), and 1 μl of PNGase F (Agilent Technologies, Catalogue number GKE-5006A). The enzyme was diluted ten times by 40 mM Tris buffer pH 7.4 to a concentration of 0.25 U/mL, and the final working concentration was 1.25 mU/mL. In this mixture, proteins were denatured and reduced, and N-glycans were enzymatically released. Following overnight incubation at 37 °C, samples were cooled down, and 600 μl MilliQ water was added. RapiGest was then cleaved with addition of 3 μl formic acid to each sample. Following incubation for 30 min at 37 °C, N-glycans were purified by solid phase extraction using Multi-Sep Hypercarb 10 mg/mL 96-well plates (Thermo Scientific, Catalogue number 60302-606, Lot 30-1090) packed with PGC on a Hamilton Starlet liquid handler equipped with a vacuum station. The columns were conditioned with 300 μl 80% acetonitrile (ACN) (Merck Millipore, Catalogue number 1.00029.2500) and 0.05% trifluoroacetic acid (TFA) (Sigma Aldrich, Catalogue number 302031-100 ML) in MilliQ water, followed by two washes with 300 μl and 200 μl of MilliQ water. The samples were then passed through the column, followed by another washing step with 300 μl MilliQ water. Finally, glycans were eluted by passing 150 μl of elution solution (40% ACN and 0.05% TFA in MilliQ water) through the columns twice. The eluate was collected in 96-well 2 mL square collection plates (Waters, Catalogue number 186002482).

After elution of glycans, the plate was dried using a vacuum concentrator with a cold trap. N-glycans were then reduced by adding 50 μl 10 mg/mL ammonium borane (Sigma Aldrich, Catalogue number 287717-10G) in MilliQ water to each well and incubating the samples for 1 h at 60 °C. Afterward, 50 μl methanol (J.T. Baker, Catalogue number 8402) was added to each well, and the plate was again dried using a vacuum concentrator. Following two wash cycles of adding 100 μl 50% methanol in MilliQ water to each well and drying the plate using a vacuum concentrator, the glycans were dissolved in 300 μl of internal standard solution (20 ng/μl maltodextrin in MilliQ water) and subjected to liquid chromatography coupled with mass spectrometry (LC-MS).

### Dextran ladder

To normalise retention times (RTs) between runs, a maltodextrin ladder was added to each sample.^52^^,^^53^ Briefly, it was prepared as follows: maltodextrin (Sigma Aldrich, Catalogue number 419699-100G) was dissolved in 100 μl MilliQ water in an Eppendorf tube to a concentration of 40 mg/mL. To reduce the glycan, 100 μl ammonium borane in water (20 mg/mL) was added to the solution, and the sample was incubated for 2 h at 60 °C. Following incubation, sample cleanup was performed by solid phase extraction using a Hypercarb 1G column (Thermo Scientific, Catalogue number 60106-403) connected to a SPE vacuum manifold. First, the column was conditioned using one volume of 80% ACN and 0.05% TFA in MilliQ water, followed by washing with two volumes of MilliQ water. Then, the maltodextrin solution was allowed to pass through the column, followed by another washing step with two volumes of MilliQ water. Finally, an elution solution consisting of 40% ACN and 0.05% TFA in MilliQ water was added to the column, and the eluate was collected and dried using SpeedVac. The dried samples were reconstituted and diluted using MilliQ water to a concentration of 200 ng/mL and were aliquoted and stored in −20 °C until use. Mass spectrometry data on the dextran ladder and conversion of RTs from minutes to normalised score is available in Table 2.

**Table 2:** List of identified molecules from the maltodextrin internal standard.

Molecule name	Molecule formula	Adduct	m/z	Charge	Retention time (min)	Normalized score
(Glc)3	C18H34O16	[M−H]	505.18	−1	7.25	0
(Glc)4	C24H44O21	[M−H]	667.23	−1	8.17	14.79
(Glc)5	C30H54O26	[M−H]	829.28	−1	8.93	25.9
(Glc)6	C36H64O31	[M−H]	991.34	−1	9.40	33.05
(Glc)7	C42H74O36	[M−H]	1153.39	−1	9.74	38.29
(Glc)8	C48H84O41	[M−H]	1315.44	−1	10.05	42.82
(Glc)9	C54H94O46	[M−2H]	738.24	−2	10.49	49.08
(Glc)9	C54H94O46	[M−H]	1477.49	−1	10.50	49.08
(Glc)10	C60H104O51	[M−2H]	820.28	−2	11.11	58.92
(Glc)10	C60H104O51	[M−H]	1640.55	−1	11.12	58.92
(Glc)11	C66H114O56	[M−2H]	900.30	−2	11.85	70.74
(Glc)11	C66H114O56	[M−H]	1801.60	−1	11.86	70.74
(Glc)12	C72H124O61	[M−2H]	981.32	−2	12.51	80.64
(Glc)13	C78H134O66	[M−2H]	1062.35	−2	12.96	87.54
(Glc)14	C84H144O71	[M−2H]	1143.38	−2	13.19	92.07
(Glc)15	C90H154O76	[M−2H]	1224.40	−2	13.01	91.51
(Glc)16	C96H164O81	[M−2H]	1305.43	−2	13.47	100

Abbreviations: Glc, glucose; m/z, mass to charge ratio.

### PGC-LC-MS

For LC-MS analysis, we used a Thermo Scientific UltiMate 3000 high-performance liquid chromatography system coupled with a Thermo Fisher Q-exactive Orbitrap mass spectrometer. For LC separation, we used a Thermo Scientific Hypercarb PGC HPLC column (150 × 1 mm, 5 μm) (Thermo Scientific, Catalogue number 35005-151030) in conjugation with a PGC trap column (20 x 2.1 mm, 7 μm). For each injection, 5 μl of the sample was injected at a rate of 100 μl/min for 3 min over the trap column and directed to waste. The rest of the gradient was directed over trap and analytical column. The mobile phases consisted of A: 10 mM ammonium bicarbonate (Sigma Aldrich, Catalogue number 09830-500G) in MilliQ water, and B: 10 mM ammonium bicarbonate in 80%/20% ACN/water (v/v). The mobile phases were applied with a flow rate of 50 μl/min as follows: 3–4 min, 95% A and 5% B; 4–15 min, 90% A and 10% B; 15–21 min, 1% A and 99% B; 21–27 min, 95% A and 5% B. For glycan detection, the MS system was running in full scan mode with negative polarity. The scan range was 500–2000 m/z, and the resolution was 35000. The heated electrospray ionisation (HESI) source was running with a spray voltage of 3 kV and a capillary temperature of 320 °C. Full LC and MS settings are found in Supplementary data. All samples were injected in a randomised order. SNAC-N samples were injected in triplicate, and the mean inter-injection CV of glycan quantification was 3.72% ± 4.47% (94.7% of CVs under 10%). Thus, the DDI samples were quantified using single injections.

In addition to full scans which were used for quantification of peaks, data dependent MS/MS was performed on all samples. Briefly, Top 5 peaks were selected for fragmentation (17,500 resolution, scan range 200–2000, normalised collision energy (NCE) 30, dynamic exclusion 10s). In order to yield fragments with more structural information for all N-glycans, additional targeted MS/MS (parallel reaction monitoring (PRM)) experiments using an inclusion list of preselected m/z values were performed with a range of NCE values (10, 20, 35 and 40), see Supplementary Information for complete MS/MS settings. The LC-settings were identical in the dd-MS2 and PRM experiments. For most of the structures, NCE 35 was optimal.

### Processing of LC-MS data

To quantify N-glycan structures, LC-MS data was imported to Skyline^54^ (version 23.1.0.455 for Windows, MacCoss Lab, University of Washington). Skyline was used in molecule interface for targeted identification of glycan structures, by comparing data with an existing list of glycan structures with specified m/z values. RTs were normalised between runs by using the added dextran ladder. Quantification was performed by automatic integration of chromatograms for each identified glycan. The automatic quantification was manually checked for each glycan and performed manually if integration had failed. Isomeric glycan structures with identical m/z values but different RTs were quantified separately. For isomeric glycans with identical m/z values, the different structures were annotated with numbers (#1, #2, etc., in ascending order according to their RT). Some peaks with large overlaps that could not be quantified separately were quantified together and annotated as such (e.g., #1–2). The integrated area of the chromatogram for each glycan of a sample was then normalised against the mean of the integrated area for the chromatogram of Glc4, Glc5, Glc6, Glc7, and Glc8 of the dextran ladder.

For definite identification of glycan structures, each identified glycan was characterised using MS2 data. MS2 spectra were imported from FreeStyle (version 1.8 for Windows, Thermo Fisher) to GlycoWorkbench^55^ (version 2.1 for Windows) updated with the latest Symbol Nomenclature for Glycans (SNFG) symbols.^56^ The MS2 spectra were annotated in GlycoWorkbench with *in-silico* matching fragments and manual inspection and knowledge of biosynthetic pathways. The MS2 spectra imported into GlycoWorkbench were from PRM experiments (NCE 35) with centroided spectra, but MS2 with lower and higher NCE levels were also compared manually. The linkage of the monosaccharides in the core N-glycan structure were assumed. For terminal structures of biological importance, such as sialic acid, bisecting GlcNAc and fucose, the position and linkage were based on chromatography and MS2. The linkage of sialic acid was determined on structures carrying only one sialic acid monosaccharide based on chromatographic separation (Siaa2,6 elute earlier than Siaa2,3) and MS2 pattern (Siaa2,3 yield more intense m/z 290 peaks with the same NCE setting).^57^ For structures with two or more sialic acid moieties, the MS2 spectra were inconclusive. Bisecting GlcNAc was assigned to a single structure with conclusive MS2 fragments. The annotation of larger high-mannose structures was inconclusive. The position of core fucose is based on unique MS2 fragments.

### Statistics

For comparison of quantitative variables between groups, we assessed groups for normality using the Shapiro–Wilk test with an alpha of 0.05. Normality was also assessed using Q–Q plots. Furthermore, homogeneity of variance was assessed using Bartlett's test with an alpha of 0.05. If all variables were normally distributed and variances were approximately equal in all compared groups, we used parametric tests such as Student's t-test for two groups or one-way ANOVA for more than two groups. If variables were not normally distributed in all compared groups, we used non-parametric tests such as the Mann-Whiney U-test for two groups, or the Kruskal–Wallis H-test for more than two groups. For comparison of qualitative variables between groups, we used Chi-squared test if the prerequisites were fulfilled (expected frequency ≥5 in each cell for two-by-two tables). If not, we used Fisher's exact test. For comparisons of single N-glycan structures between groups, the p-value was adjusted for multiple comparisons using the Holm-Šidak method. Correlations between N-glycan structures were assessed using Spearman's r, as N-glycan levels were not normally distributed. All statistical tests used in this study were two-tailed. A significance level of 0.05 was used.

For clustering analysis, N-glycan levels for each individual were first z-standardised by subtracting with the mean with the specific N-glycan and dividing with the standard deviation. The resulting z-score was used as a measure of abundance of a specific glycan in each sample. Unsupervised hierarchical clustering was used to identify outlier samples. We performed unweighted pair group method with arithmetic mean (UPGMA), a bottom-up clustering method, using the hclust function in R. For dimensionality reduction of the data, we also performed t-SNE using the “Rtsne”^58^ package in R. For t-distributed stochastic neighbour embedding (t-SNE) analysis, we used the settings dimensions = 2, perplexity = 5, and iterations = 1000, with no initial principal component analysis (PCA) step. A low perplexity setting was used as the cohorts were relatively small. Heatmaps were created using the “pheatmap”^59^ package.

To evaluate if low blood N-glycosylation could predict future cognitive decline or biomarker changes, we applied linear mixed effects models on longitudinal cognitive and biomarker data. For this purpose, we used the R-packages “lme4”^60^ and “lmerTest”.^61^ The outcome variable was either CSF biomarker levels (Aβ42/40, pTau181 and t-tau, in pg/mL) or cognitive test scores (CERAD recall, MMSE and TMT-B, in points on the scales). We included an individual-level random effect, which was assumed to follow a normal distribution. In order to determine if the CSF biomarker or cognitive test score trajectories were significantly different in the low blood N-glycosylation group and the rest of the cohort, the interaction term between time (in years) and low blood N-glycosylation (dichotomised as positive/negative) was used as the predictor variable. Other predictor variables were age, sex, length of education, and *APOE4* allele status. The continuous variables age and length of education were z-standardised before analysis. The assumptions underlying linear mixed-effects models with random intercepts were tested in the following way: linearity was assessed by plotting observed data against fitted values. Normality of residuals and random effect was assessed with Q–Q plots. Homoscedasticity was tested by plotting residuals vs. fitted values. T-tests for significance were performed according to Satterthwaite's method. Profile likelihood-based 95% confidence intervals were calculated for all predictor variables. Data wrangling was performed using “readxl”,^62^ “tidyr”,^63^ and “dplyr”^64^ packages. Graphing was performed using “ggplot2”^65^ and “ggeffects”^66^ packages.

All statistical analyses were performed in GraphPad Prism, version 10.1.2 for Windows (www.graphpad.com; GraphPad Software, San Diego, California USA) or R version 4.4.0 (r-project.org; R Foundation for Statistical Computing, Vienna, Austria). Figures were created using Adobe Illustrator.

### Role of funders

In the present study, no funding source had any role in study design, data collection, data analyses, interpretation, or writing of this report.

## Results

### The blood N-glycome is similar between cohorts

**Fig. 1:**
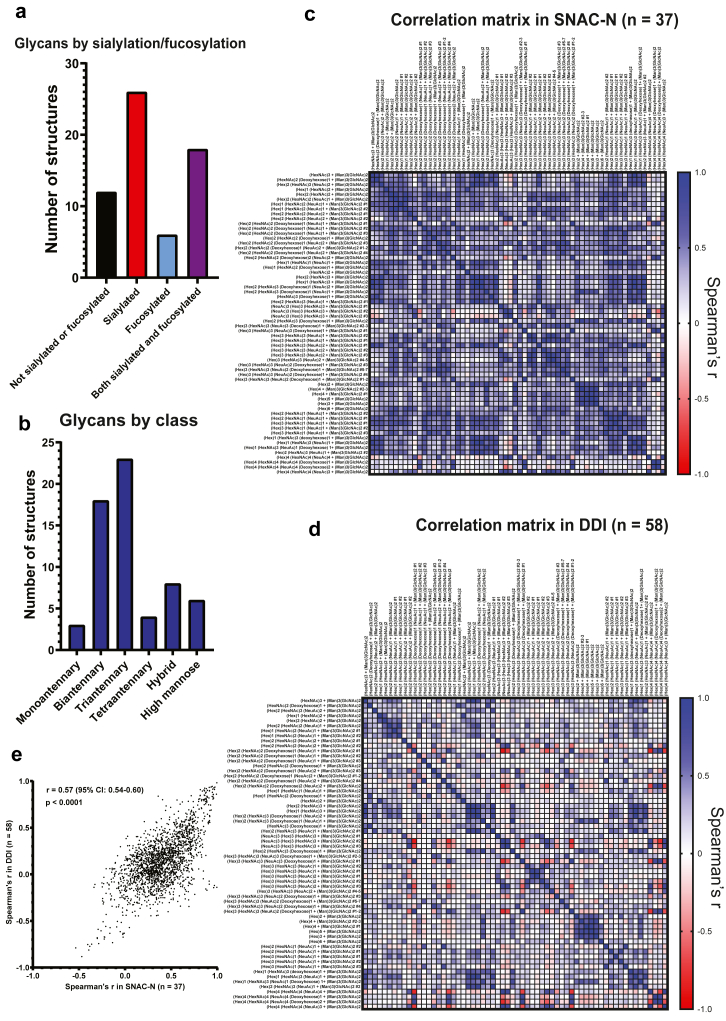
**Summary of N-glycan structures identified in blood. (a)** Bar chart showing the frequency of sialylation/fucosylation in detected N-glycans in blood. **(b)** Bar chart showing number of glycans in each structural category identified in this study. **(c)** Correlation matrix (Spearman correlation) of all identified N-glycans in the SNAC-N study (n = 37). **(d)** Correlation matrix (Spearman correlation) of all identified N-glycans in the DDI study (n = 58). **(e)** Scatterplot showing Spearman's r correlation coefficients between each combination of blood N-glycans in the SNAC-N study (x-axis) vs. in the DDI study (y-axis). In (c–d), colours represent Spearman's r correlations between glycans. Numbers after glycan structures indicate different isomers ranked by retention times (numbered from low to high).

In total, we identified 62 unique N-glycan structures, separated by LC-MS by either m/z, RTs, or both. A list of all identified structures based on monosaccharide composition can be found in Supplemental Table S1. Representative chromatograms of all glycan structures grouped by structural class are shown in Supplementary Fig. S1. We use the following abbreviations for monosaccharides in this paper: GlcNAc, N-acetylglucosamine; Hex, hexose; HexNAc, N-acetylhexosamine; Man, mannose; NeuAc, neuraminic acid.^67^ Most glycans in blood were sialylated, with the most abundant glycan being the biantennary sialylated glycan (Hex)2 (HexNAc)2 (NeuAc)2 + (Man)3 (GlcNAc)2 (m/z 1111.39). Fig. 1a and b summarise identified glycans based on fucosylation/sialylation and structural class.

Before further analysis, we used unsupervised hierarchical clustering to identify outlier samples. In the Swedish SNAC-N cohort (n = 40), one sample was excluded from further analysis since only a few N-glycans were detected. Another two outlier samples were excluded through hierarchical clustering (Supplemental Fig. S2a). In the Norwegian DDI cohort (n = 60), two outlier samples were excluded through hierarchical clustering (Supplemental Fig. S2b).

Next, we compared the blood N-glycome in the SNAC-N cohort with the DDI cohort and determined the relative expression of each blood N-glycan structure. Within each cohort, we calculated the Spearman's r correlation coefficient between all possible combinations of glycans. Correlation matrices between all glycan structures in both cohorts are shown in Fig. 1c and d. We then compared these for each combination of glycans in the SNAC-N cohort and the DDI cohort (Fig. 1e) and found a highly significant correlation (Spearman's r = 0.57 [95% confidence interval (CI): 0.54–0.60], p < 0.0001). Thus, the relative distribution of the N-glycans is similar in the two cohorts. Given that the two populations are different (Swedish vs. Norwegian, older vs. younger, includes AD cases vs. only SCD or MCI) a perfect correlation is not expected. Since we analysed plasma samples in the SNAC-N cohort and serum samples in the DDI cohort, the clear correlation between the two groups suggests that there are no major differences in the glycan pattern in plasma compared to serum.

### Reduced levels of blood N-glycans was associated with AD

**Fig. 2:**
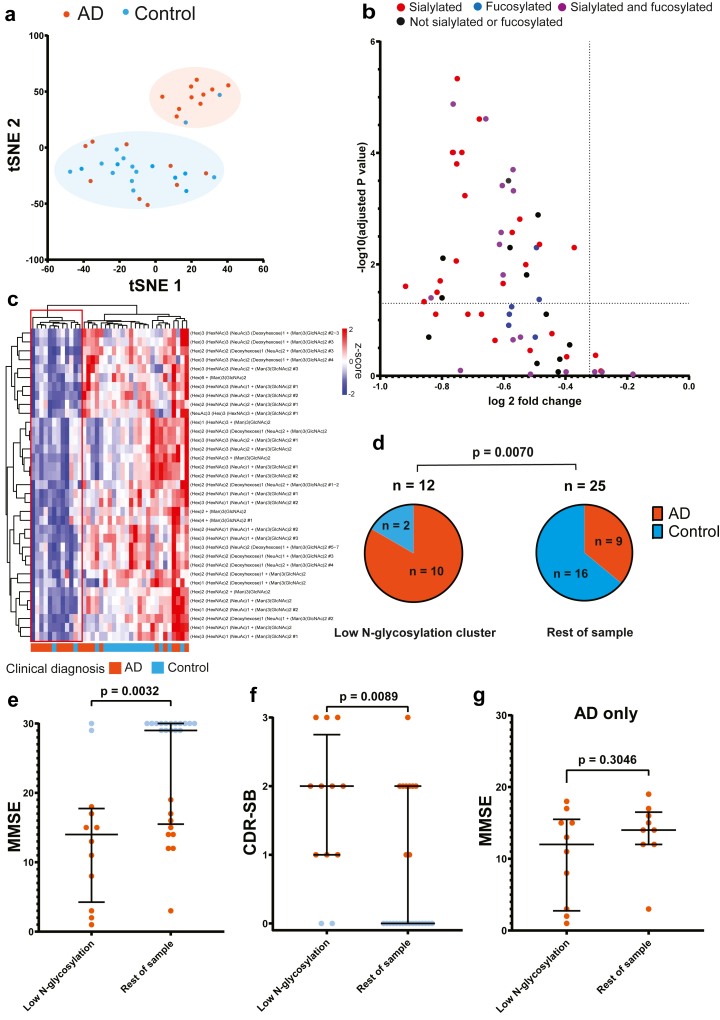
**A subset of patients with AD exhibited reduced N-glycosylation in the SNAC-N cohort. (a)** t-distributed stochastic neighbour embedding (t-SNE) plot showing two distinct clusters in SNAC-N (n = 37), with one cluster enriched in patients with AD. We included all measured glycans and applied t-SNE with perplexity = 5 and iterations = 1000. **(b)** Volcano plot of all glycan structures when comparing the previous AD-enriched cluster (n = 12) with the rest of the SNAC-N cohort (n = 25). P-values were calculated by applying the Mann–Whitney U-test, and correction for multiple comparisons were performed using the Holm-Sidak method. **(c)** Heatmap of N-glycan values in SNAC-N, only including the glycans that were significantly altered in (b). **(d)** Pie chart showing the distribution of AD cases in the low N-glycosylation cluster (n = 12) compared to the rest of the cohort in SNAC-N (n = 27). There were a larger portion of AD cases within the low N-glycosylation group compared to the rest of the cohort (p = 0.0070, Chi-squared test). **(e)** Mini-Mental State Examination (MMSE) scores were lower in the low N-glycosylation group compared to the rest of the cohort (p = 0.0032, Mann–Whitney U-test). **(f)** Clinical Dementia Rating—Sum of Boxes (CDR-SB) scores were higher in the low N-glycosylation group compared to the rest of the cohort (p = 0.0089, Mann–Whitney U-test). **(g)** MMSE scores were not significantly altered in AD patients with low N-glycosylation compared to AD patients without low N-glycosylation (p = 0.30, Mann–Whitney U-test). In (e–g), long horizontal lines indicate median values, error bars indicate interquartile ranges, red dots indicate individuals with AD, and blue dots indicate control individuals.

The remaining samples from the SNAC-N study (n = 37) were analysed with clustering methods. Before analysis, the level of each glycan structure in one individual was z-standardised by subtracting the mean of the specific glycan structure across the cohort and dividing the result by the standard deviation of the glycan. T-SNE performed with perplexity = 5 and iterations = 1000 revealed two distinct clusters (Fig. 2a), with one cluster enriched in AD cases. Compared to the rest of the cohort, this cluster of 12 individuals (32.4% of cohort) had significantly lower levels of a wide range of N-glycans (Fig. 2b). Unsupervised hierarchical clustering with the significantly altered glycans in this cluster compared to the rest of the cohort similarly identified AD-enriched clusters (Fig. 2c). The low N-glycan cluster consisted of 10 AD cases and 2 control cases, while the rest of the cohort included 9 AD cases and 16 control cases (83% AD vs. 36% AD; χ^2^: p = 0.0070) (Fig. 2d).

Since the AD diagnosis in the SNAC-N cohort was based on clinical assessment, we next measured the levels of pTau217 and GFAP in blood to assess if individuals with low N-glycosylation also showed evidence of biological AD pathology and neuroinflammation. Individuals with low N-glycosylation had higher levels of blood pTau217 compared to the rest of the cohort (Supplemental Fig. S3a) (low N-glycosylation: median = 0.57 pg/mL, interquartile range (IQR) = 0.20–0.91; rest of cohort: median = 0.16 pg/mL, IQR = 0.09–0.31; Mann Whitney U-test: p = 0.0036). GFAP was elevated in the low N-glycosylation group compared to the rest of the cohort, though the difference was not significant (Supplemental Fig. S3b) (low N-glycosylation: median = 119.4 pg/mL, interquartile range (IQR) = 86.1–200.0; rest of cohort: median = 90.1 pg/mL, IQR = 55.0–127.3; Mann Whitney U-test: p = 0.057).

When comparing cognitive performance between individuals in the low N-glycosylation group to the rest of the cohort, the low N-glycosylation group had lower scores in the MMSE test (Fig. 2e) (low N-glycosylation: median = 14, IQR = 4.25–17.75; rest of cohort: median = 29, IQR = 15.5–30; Mann Whitney U-test: p = 0.0032) and had worse CDR-SB (Fig. 2f) (low N-glycosylation: median = 2, IQR = 1–2.75; rest of cohort: median = 0, IQR = 0–2; Mann Whitney U-test: p = 0.0089). Within the AD group, individuals with low N-glycosylation had lower MMSE scores, though the difference was not significant (Fig. 2g) (low N-glycosylation: median = 12, IQR = 2.75–15.5; rest of cohort: median = 14, IQR = 12–16.5; Mann Whitney U-test: p = 0.30).

### Low blood N-glycosylation was associated with APOE4 but not with amyloid/tau in a younger population

**Fig. 3:**
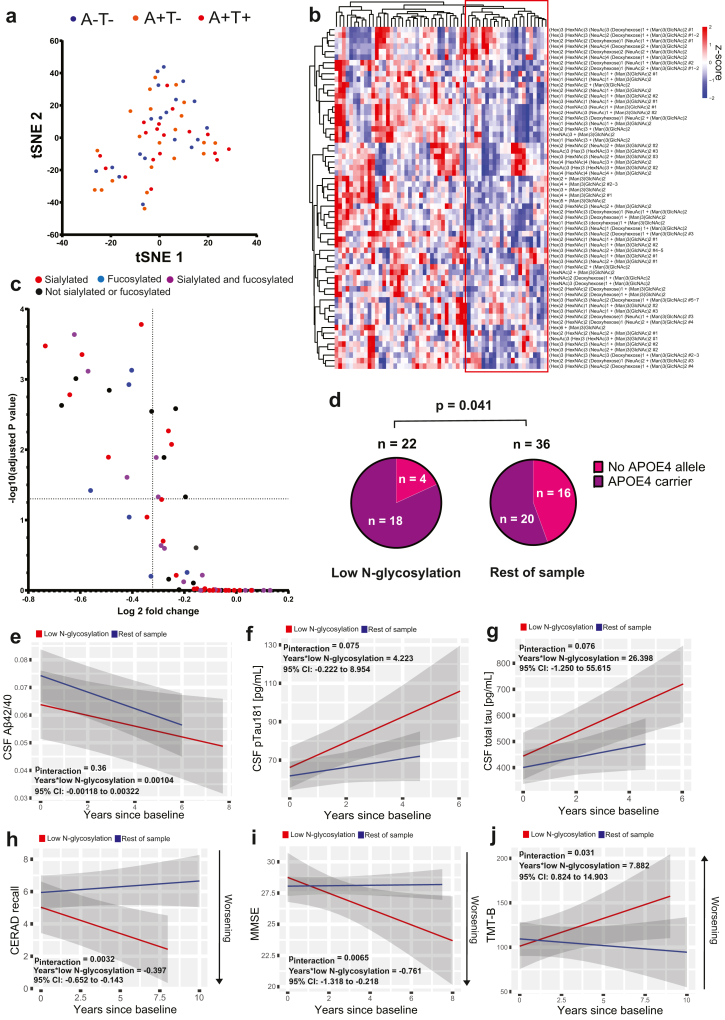
**Low N-glycosylation was associated with *APOE4* and cognitive decline in the DDI cohort. (a)** t-distributed stochastic neighbor embedding (t-SNE) plot when applying t-SNE with perplexity = 5 and iterations = 1000 on the DDI cohort (n = 58). Individuals are coloured by amyloid/tau (A/T) status. **(b)** Heatmap with unsupervised hierarchical clustering revealing a cluster with low N-glycosylation (red box, n = 22). **(c)** Volcano plot of all N-glycans when comparing the low N-glycosylation cluster (n = 22) to the rest of the DDI cohort (n = 36). p-values were calculated by applying the Mann–Whitney U-test, and correction for multiple comparisons were performed using the Holm-Sidak method. **(d)** Pie chart showing the frequency of *APOE4* carriers in the low N-glycan cluster (n = 22) compared to the rest of the DDI cohort (n = 36). *APOE4* carriership was more common in the low N-glycan group compared to the rest of the cohort (p = 0.0041, Chi-squared test). **(e**–**g)** Longitudinal trajectories of cerebrospinal fluid (CSF) biomarkers in the low N-glycosylation cluster (n = 22) compared to the rest of the cohort (n = 36). The groups did not differ in longitudinal CSF Aβ42/40 ratio (p = 0.36, Satterthwaite t-test), pTau181 (p = 0.075, Satterthwaite t-test), or t-tau (p = 0.076, Satterthwaite t-test). **(h**–**j)** Longitudinal trajectories of cognitive test scores in the low N-glycosylation cluster (n = 22) compared to the rest of the cohort (n = 36). Individuals with low N-glycosylation showed faster cognitive decline according to CERAD recall score (p = 0.0032, Satterthwaite t-test), MMSE score (p = 0.0065, Satterthwaite t-test), and TMT-B score (p = 0.031, Satterthwaite t-test). Graphs in (e–j) were created using the “ggplot2” and “ggeffects” packages in R. Shaded areas indicate 95% confidence bands.

Next, we validated our findings by analysing samples from the Norwegian DDI cohort, consisting of younger individuals characterised with amyloid/tau (A/T) biomarkers. Our goal was to evaluate whether the low N-glycosylation group could be identified in younger individuals at earlier clinical and biological stages of the disease. After exclusion of two outlier samples, serum samples from 58 A/T-characterised individuals from the DDI cohort were analysed with identical clustering methods as described above. When we applied t-SNE on N-glycomic data in the DDI cohort with identical settings as described previously, individuals did not separate according to A/T status (Fig. 3a). When unsupervised hierarchical clustering was applied, we identified a subgroup with reduced N-glycosylation consisting of 22 individuals (37.9% of cohort) (Fig. 3b). Compared to the rest of the cohort, levels of 22 N-glycans were significantly reduced in this group (Fig. 3c). In this low glycan group in the DDI cohort, 18 of 22 individuals were *APOE4* carriers. In the rest of the cohort, 20 of 36 individuals were *APOE4* carriers (81.8% in low glycan group vs. 55.6% in rest of cohort; χ^2^: p = 0.041) (Fig. 3d). The low N-glycan group did not differ significantly from the rest of the cohort when comparing CSF levels of Aβ42/40 ratio, pTau181, or t-tau (Supplemental Fig. S4).

### Low blood N-glycosylation was associated with future cognitive decline

Next, we evaluated if low blood N-glycosylation could predict cognitive decline or amyloid/tau accumulation. We employed linear mixed effects models with individual-level random effects. The dependent outcome was either CSF biomarker level (Aβ42/40, pTau181 or t-tau) or cognitive test score. The cognitive tests used were the CERAD word list recall test, MMSE, and TMT-B. The interaction term between years elapsed and low N-glycosylation was evaluated.

Low blood N-glycosylation was not significantly associated with longitudinal changes of Aβ42/40, pTau181, or total tau (Fig. 3e–g). However, individuals in the low N-glycosylation group consistently exhibited faster cognitive decline compared to the rest of the cohort (Fig. 3h–j). In the CERAD recall test, individuals with low N-glycosylation declined faster by 0.40 points per year (estimate [β]: −0.40 [95% CI: −0.65 to −0.14], p = 0.0032). In the MMSE test, individuals with low N-glycosylation declined faster by 0.76 points per year (β: −0.76 [95% CI: −1.32 to −0.22], p = 0.0065). Finally, in the TMT-B test, individuals with low N-glycosylation declined faster by 7.9 points per year (β: 7.9 [95% CI: 0.82–14.90], p = 0.031). Importantly, in all cognitive tests, individuals without low N-glycosylation remained stable throughout the follow-up period. All estimated parameters of linear mixed-effects models are available in Supplemental Tables S2 and S3.

As a sensitivity analysis, we adjusted the linear models for age, sex, length of education, and *APOE4* genotype, none of which affected the predictive value of low N-glycosylation. To determine whether the glycan effect on cognitive decline was independent of CSF amyloid/tau biomarker status, we also adjusted the linear models with cognitive performance as the outcome variable by adding baseline A/T status as predictor variables. This adjustment did not alter the estimated effect of low N-glycosylation. Furthermore, baseline A/T positivity was associated with worse baseline cognition, but not with worse rate of cognitive decline (Supplemental Fig. S5). Taken together, the results suggest that low baseline N-glycosylation predicts rate of cognitive decline in an amyloid and tau independent manner.

### A wide range of glycans are less abundant in the low N-glycosylation group

**Fig. 4:**
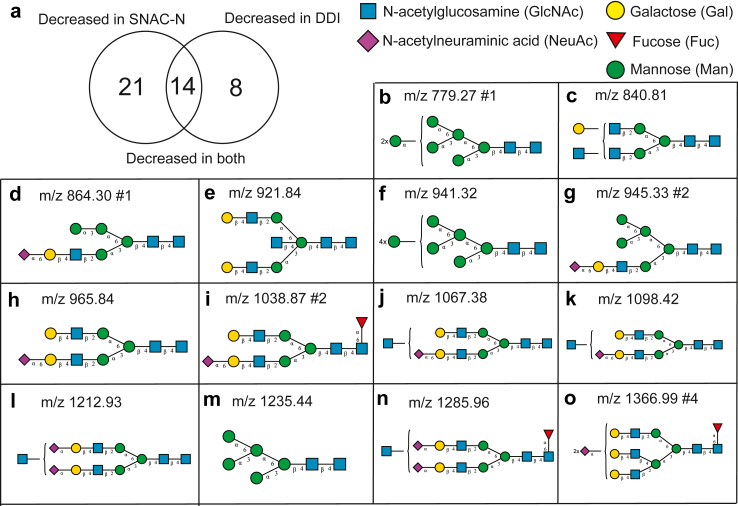
**Significantly reduced blood N-glycans in the low N-glycosylation group. (a)** Pie chart showing number of significantly reduced blood N-glycans in the low N-glycosylation group in the SNAC-N cohort and DDI cohort. Fourteen N-glycans were reduced in low blood individuals with low N-glycosylation in both cohorts. **(b**–**o)** Putative structures of significantly reduced glycan structures, and their corresponding mass to charge (m/z) ratios. Isomeric glycans with identical m/z ratios but different retention times are annotated with the peak number ordered from low to high retention time (e.g., peak #1). Structures were visualised using GlycoWorkbench. N-glycans were drawn according to the Symbol Nomenclature for Glycans (SNFG).

In total, the levels of 14 N-glycan structures in blood were significantly reduced in the low N-glycosylation group of both the SNAC-N and DDI cohorts (Fig. 4a), and could be further explored as a biomarker for cognitive decline in AD. Using MS2 data, we elucidated the structure of these 14 glycans. Putative structures for each of these 14 glycans are shown in Fig. 4b–o. They include a wide range of glycans, including sialylated glycans, fucosylated glycans, high-mannose structures, and several types of complex structures. Although only 14 specific glycan structures were significantly decreased in the low N-glycosylation group in both cohorts, the total level of all measured blood N-glycans was also significantly lower in the low N-glycosylation group compared to the rest of the cohort (Supplemental Fig. S6).

## Discussion

In this study, we used N-glycomics and showed that a subset of individuals (32.4% of the SNAC-N cohort and 37.9% of the DDI cohort) had reduced blood N-glycosylation. In the younger DDI cohort from Norway, low blood N-glycosylation was prevalent in *APOE4* carriers and predicted cognitive decline in an amyloid-independent manner. In the older SNAC-N cohort from Sweden, low blood N-glycosylation was associated with worse cognition and clinical/biological AD disease. In summary, we present 14 N-glycan structures as candidate biomarkers in blood for cognitive decline.

Our findings in the two cohorts are congruent with each other. In the younger DDI cohort, where all individuals included in this study were dementia-free at baseline, low blood N-glycosylation was associated with worse future cognitive decline. In the older SNAC-N cohort, low blood N-glycosylation was associated with worse cognition, elevated blood pTau217, and dementia. Our results are in line with Frenkel-Pinter et al.,^68^ who found lower levels of protein glycosylation in blood in patients with AD compared to controls using periodic acid-Schiff staining and lectin blotting. Similarly, Chen et al. previously found lower levels of one specific core-fucosylated biantennary N-glycan in blood in patients with AD compared to controls.^37^

Previous studies on N-glycosylation in AD brain have revealed several changes, such as reduced levels of sialylated glycans.^19^^,^^21^ Two further studies in CSF,^24^^,^^38^ and one study in blood, found reduced levels of sialylated glycans.^68^ This relates to our findings in blood, where we found reduced levels of several sialylated glycans in the low N-glycosylation risk group for dementia. However, it is unknown whether changes in N-glycosylation in blood reflect changes in brain N-glycosylation in AD. Future studies using paired CSF/blood-samples might shed light on this matter.

In the low N-glycosylation risk group for AD, we found decreased levels of a wide range of glycans of various structural types. This is unlikely to be caused by defects or dysfunctions in single catalytic glycosyltransferase enzymes. Indeed, one previous study found that expression of several genes involved in pivotal steps of cellular N-glycan synthesis were downregulated in AD,^69^ which would be consistent with a general decrease of N-glycan levels. Furthermore, we suggest that several other pathogenic processes in AD–including endoplasmic reticulum dysfunction,^70^ Golgi dysfunction,^71^^,^^72^ or dysregulated systemic inflammation^73^–could cause disruption of protein N-glycosylation and thus lead to the low N-glycosylation phenotype that we observed. Altered or reduced glycosylation is expected to lead to many pathological processes in AD, such as dysfunctional endolysosomal activity.^74^^,^^75^

We note that low blood N-glycosylation was not associated with CSF amyloid or tau biomarkers and did not predict future reduction of CSF Aβ42/40 in the DDI cohort. There was a trend of faster increase of both CSF pTau181 and total tau in the group with low blood N-glycosylation, though the difference between groups was not significant. On the contrary, low blood N-glycosylation was highly correlated with cognitive decline. We propose that low blood N-glycosylation is a risk factor for cognitive decline due to AD, irrespective of their CSF amyloid or tau levels. Alternatively, low blood N-glycosylation could be a biomarker of non-AD-specific neurodegeneration. However, with a prevalence of over 30% in mixed cohorts of cognitively healthy controls and clinical/biological AD, we consider it implausible that low blood N-glycosylation represents a different dementia disease. Indeed, low blood N-glycosylation was associated with the AD risk gene *APOE4* in the DDI cohort. Additionally, individuals with low N-glycosylation had higher levels of blood pTau217 in the older SNAC-N cohort, which includes cognitively impaired individuals with clinical AD diagnosis. Still, further studies are needed to determine the disease specificity of this phenotype.

Our study has several important strengths. First, we used a robust and reproducible N-glycan quantification method to evaluate blood N-glycosylation as an AD biomarker in well-characterised dementia research cohorts. Furthermore, we validated our findings in a cohort that is very different regarding age and disease stages compared to the discovery cohort. We showed that low N-glycosylation was associated with cognitive decline in both cohorts, using a range of different cognitive tests. As we evaluated blood N-glycosylation in a population-based cohort (SNAC-N), the results may be more applicable in unselected populations, because memory clinic cohorts tend to be less representative of the general population.^76^ Finally, the disease groups in both cohorts were matched by age and sex (and education in the DDI cohort) to eliminate possible confounders for blood N-glycome analysis.

Our study also has weaknesses. In this study, no pre-analytical power calculation was performed, and the participants were selected from larger cohorts, thus introducing the risk for selection bias. Additionally, although we attempted to control for confounding factors, many possible confounders regarding cognitive decline were not measured and could not be controlled for, such as cardiovascular disease burden. Finally, the 95% confidence intervals for some estimates in our analyses are wider than ideal, reflecting the limitations of our sample size. This reduced precision may increase uncertainty in the effect sizes and associations reported. Therefore, our findings should be further validated in larger and unselected cohorts to eliminate confounding factors and improve the precision of estimates. Furthermore, individuals with non-Caucasian ethnicities were underrepresented in this study, affecting the generalisability of the conclusions.

The advent of AD-specific therapies leads to greater needs for prognostic biomarkers that will inform clinicians of which individuals are at greatest risk for cognitive decline. Novel blood-based biomarkers can detect biological AD changes defined as cerebral amyloidosis with very high accuracy.^28^^,^^77^ Still, a large portion of amyloid positive individuals do not develop dementia in their lifetime and may not need intervention.^78^^,^^79^ To this end, other modalities can be used to identify individuals at risk for dementia development, most notably Tau Positron Emission Tomography (Tau-PET).^80^ In this paper, we present a novel way to detect individuals at higher risk for cognitive decline due to AD, using only a blood test. Since the blood N-glycosylation pattern differed within the clinical/biological AD groups, we suggest that blood N-glycome profiling could be used to divide the AD population into different subgroups with varying risk for disease progression. Furthermore, aberrant glycosylation is increasingly seen as a potential target for AD therapeutics. Inhibition of O-GlcNAcase is being evaluated as an AD treatment,^81^ and reversal of aberrant N-glycosylation can also attenuate AD pathology in animal models.^82^ Thus, blood N-glycome profiling also has potential for patient selection and monitoring for future glycosylation-targeting therapies for AD.

In conclusion, we present a robust blood N-glycome profiling method that could be applied to AD or other diseases, such as cancer or other inflammatory conditions.^31^ Reduced blood N-glycosylation was associated with cognitive decline and AD in older individuals, and *APOE4* allele carriership and future cognitive decline in younger individuals. Thus, we suggest that blood N-glycosylation is a promising prognostic biomarker in AD and could be used in future precision-medicine approaches to identify at-risk individuals for cognitive decline. Still, further validation in larger cohorts is needed to support the use of blood N-glycosylation as a routine AD biomarker.

## Contributors

RZZ: conceptualisation, data curation, formal analysis, funding acquisition, investigation, software, validation, visualisation, writing–original draft, writing—review & editing, accessed and verified the underlying data; SG: conceptualisation, data curation, formal analysis, funding acquisition, investigation, methodology, software, supervision, validation, visualisation, writing—original draft, writing—review & editing, accessed and verified the underlying data; BEK: data curation, formal analysis, funding acquisition, resources, writing—review & editing; BL: investigation, validation; MH: investigation, validation; AJ: data curation, investigation, validation; AS: data curation, resources, writing—review & editing; AW: data curation, resources, writing—review & editing; BW: funding acquisition, project administration, supervision; TF: data curation, funding acquisition, resources, writing—review & editing; SSW: conceptualisation, data curation, funding acquisition, methodology, project administration, resources, supervision, writing—review & editing; LOT: conceptualisation, funding acquisition, methodology, project administration, resources, supervision, writing—review & editing. All authors read and approved the final version of the manuscript.

## Data sharing statement

Anonymised LC-MS raw data used to generate the results in this article is available through the database of Open Science Framework (osf.io/8ucsm). Clinical data from the DDI cohort utilised in this project is housed at the Services for Sensitive Data (TSD) at the University of Oslo (UiO) and is not publicly accessible. Nonetheless, anonymised data from both DDI and SNAC-N cohorts can be provided by the corresponding author upon a reasonable request.

## Code sharing

The R code which was used to perform analyses in this study is available for download as a supplementary file.

## Declaration of interests

AW is a license holder of the Resource Utilisation in Dementia (RUD)-instrument and is a member of ADI Medical and Scientific Advisory Panel (ADI MSAP). B.E.K. has served as a consultant for Biogen and at the advisory board for Eisai and Eli Lilly. T.F. has served as a consultant and at the advisory boards for Biogen, Novo Nordisk, Eli Lilly, Bioarctic and Roche. TF also received payment from Bioarctic for lectures. TF has stock options in Pre Diagnostic AS. All other authors have no conflicts of interest to disclose.

## Appendix A Supplementary data

Glycomics project.R

Supplementary Figures

Supplemental methods_LC settings

Supplemental methods_MS settings

Supplemental methods_MS2 settings NCE10

Supplemental methods_MS2 settings NCE20

Supplemental methods_MS2 settings NCE35

Supplemental methods_MS2 settings NCE40
