# Supplementary material for: Blood N-glycomics reveals individuals at risk for cognitive decline and Alzheimer’s disease
Source: eBioMedicine. 2025 Feb 20;113:105598. doi: 10.1016/j.ebiom.2025.105598 (PMC11893330; doi:10.1016/j.ebiom.2025.105598)
Supplement: Supplementary Figures [file mmc2.docx]

**Supplement to: Blood N-glycomics reveals individuals at risk for cognitive decline and Alzheimer’s disease.**

**Contents**

[**Figure S1. Representative extracted ion peak chromatograms of LC-MS quantification of N-glycans.** 2](#_Toc184227417)-3

[**Figure S2. Identification of outliers with unsupervised hierarchical clustering.** 4](#_Toc184227418)

[**Figure S3. Blood biomarkers in SNAC-N cohort stratified by plasma N-glycan levels.** 5](#_Toc184227419)

[**Figure S4. Cerebrospinal fluid (CSF) biomarker levels in the Dementia Disease Initiation cohort stratified by serum N-glycan levels.** 6](#_Toc184227420)

[**Figure S5. Longitudinal trajectory of cognitive test scores stratified by baseline amyloid/tau status.** 7](#_Toc184227421)

[**Figure S6. The total level of blood N-glycans in the low N-glycosylation cluster compared to the rest of the sample** 8](#_Toc184227422)


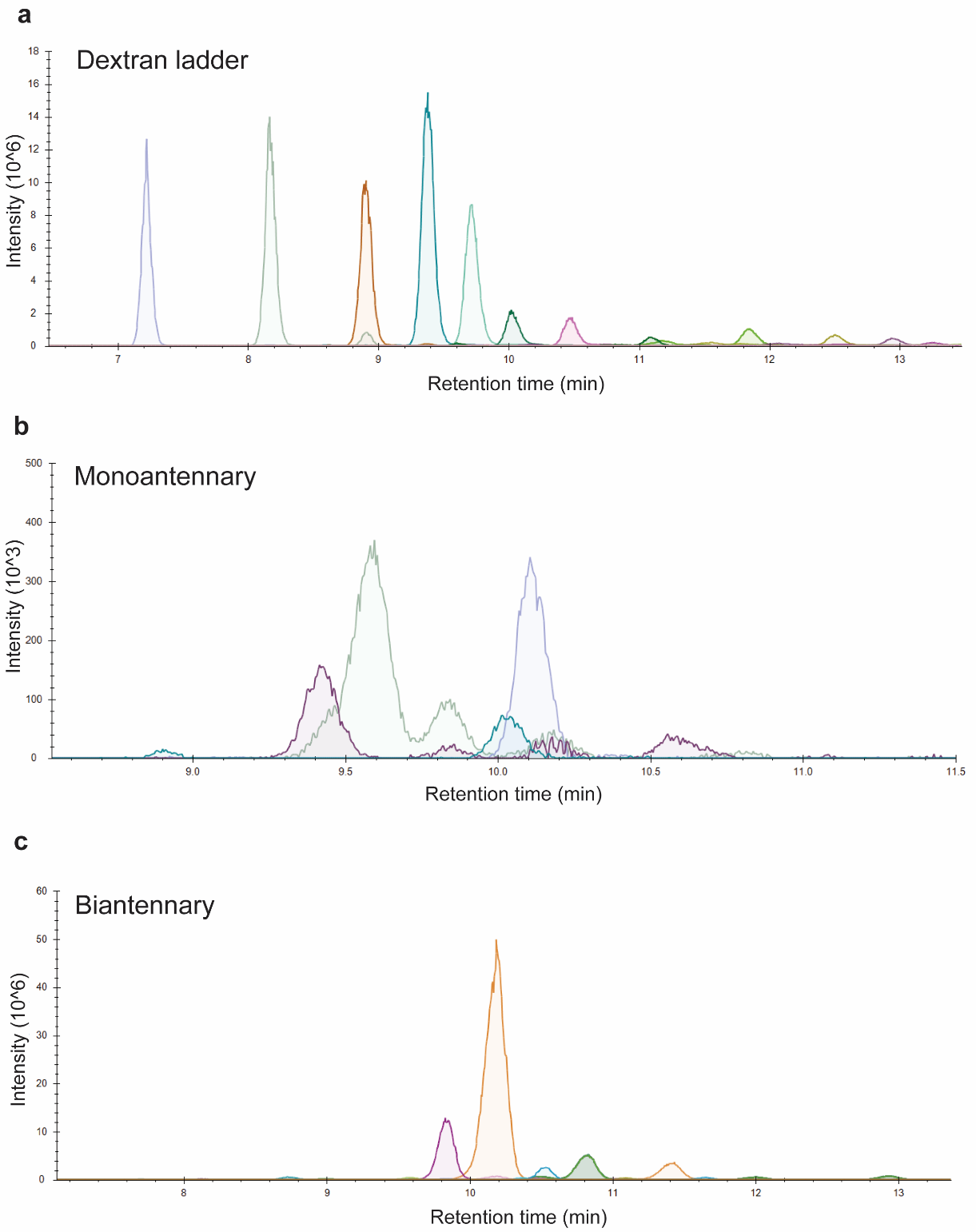


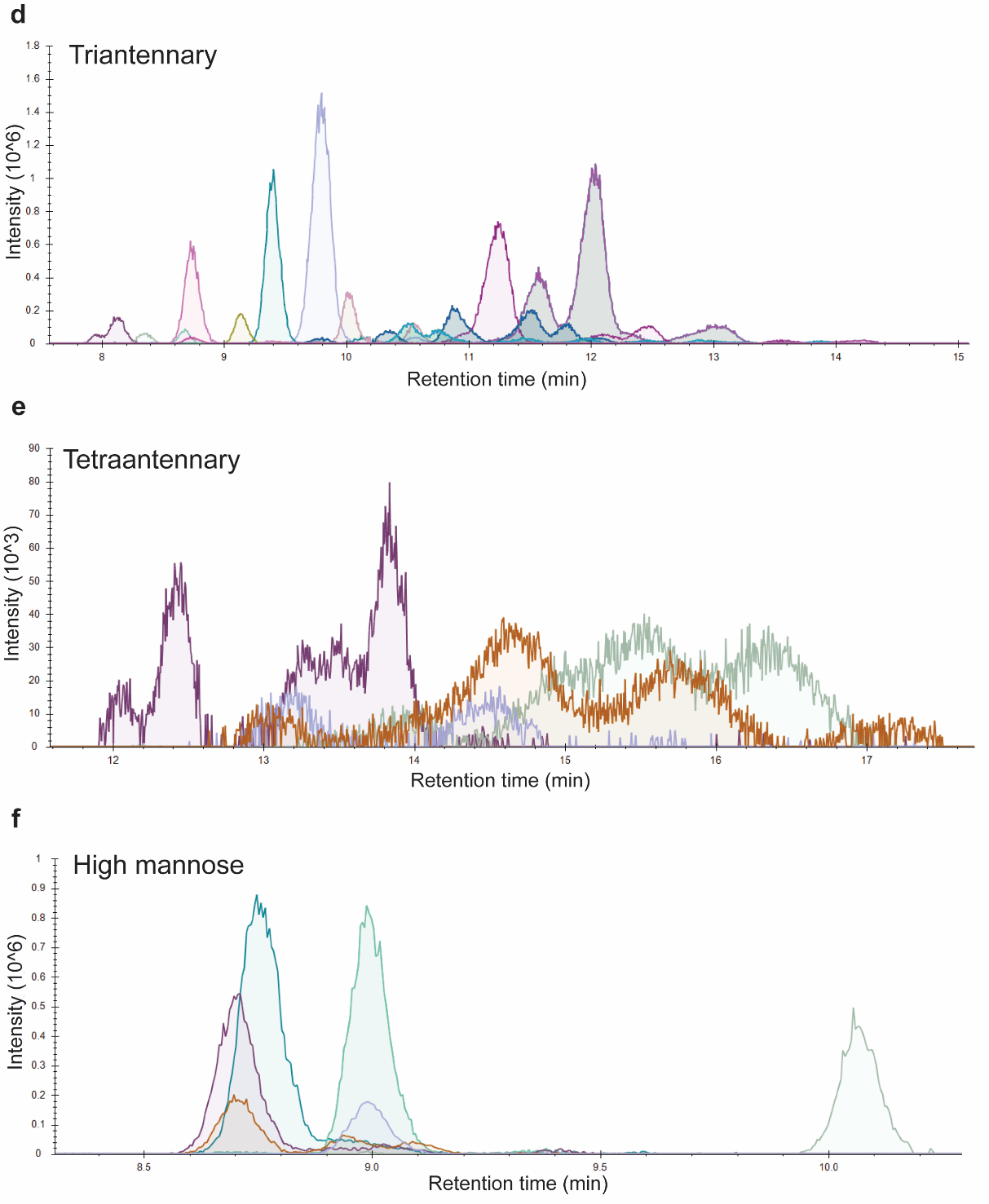


*contd.*


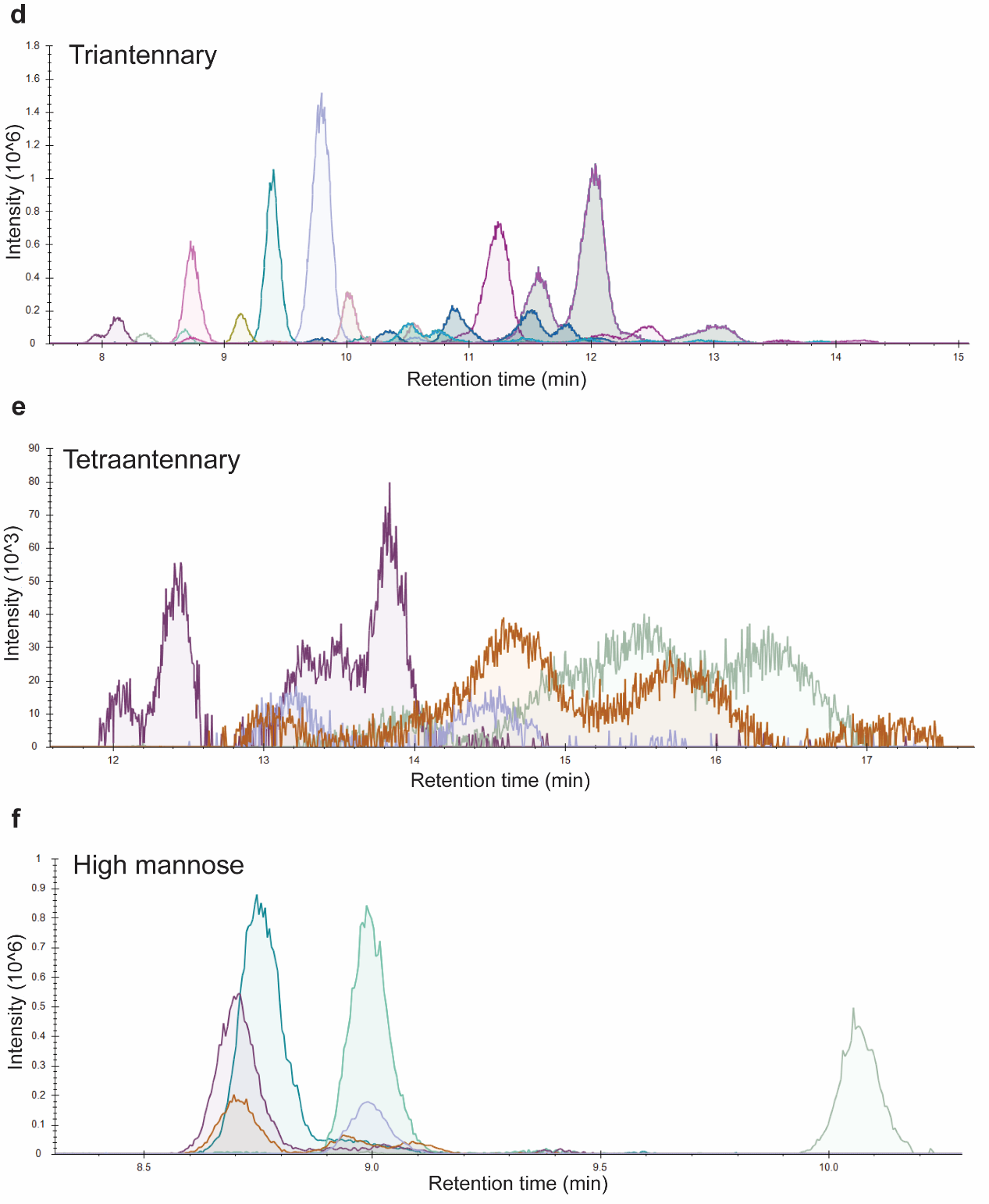


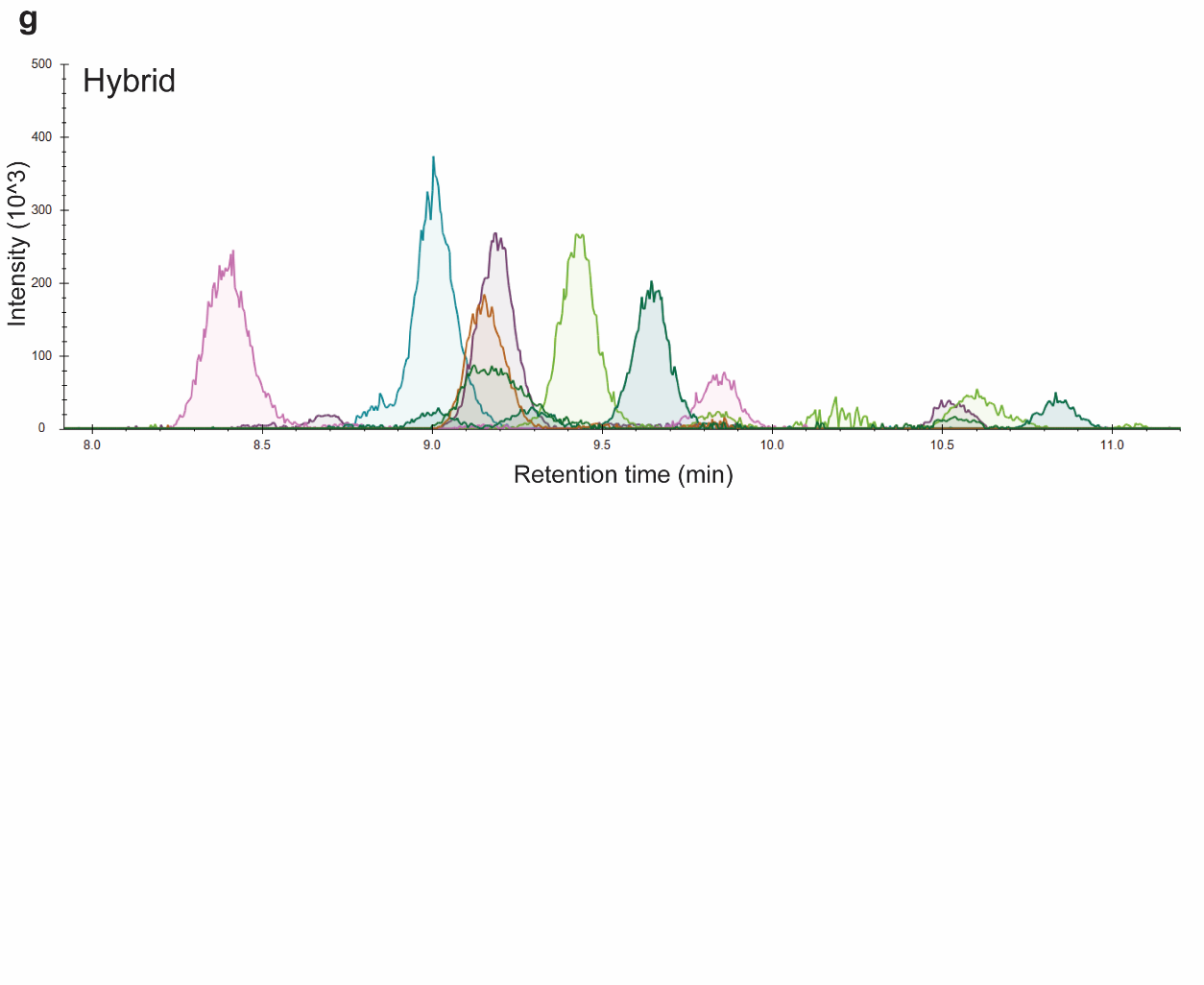


**Figure S1. Representative extracted ion peak chromatograms of LC-MS quantification of N-glycans.** Each chromatogram contains glycans of a certain structural class: **(a)** dextran ladder standard, **(b)** monoantennary glycans, **(c)** biantennary glycans, **(d)** triantennary glycans, **(e)** tetraantennary glycans, **(f)** high mannose glycans, **(g)** hybrid structures. Intensity is measured in arbitrary units.


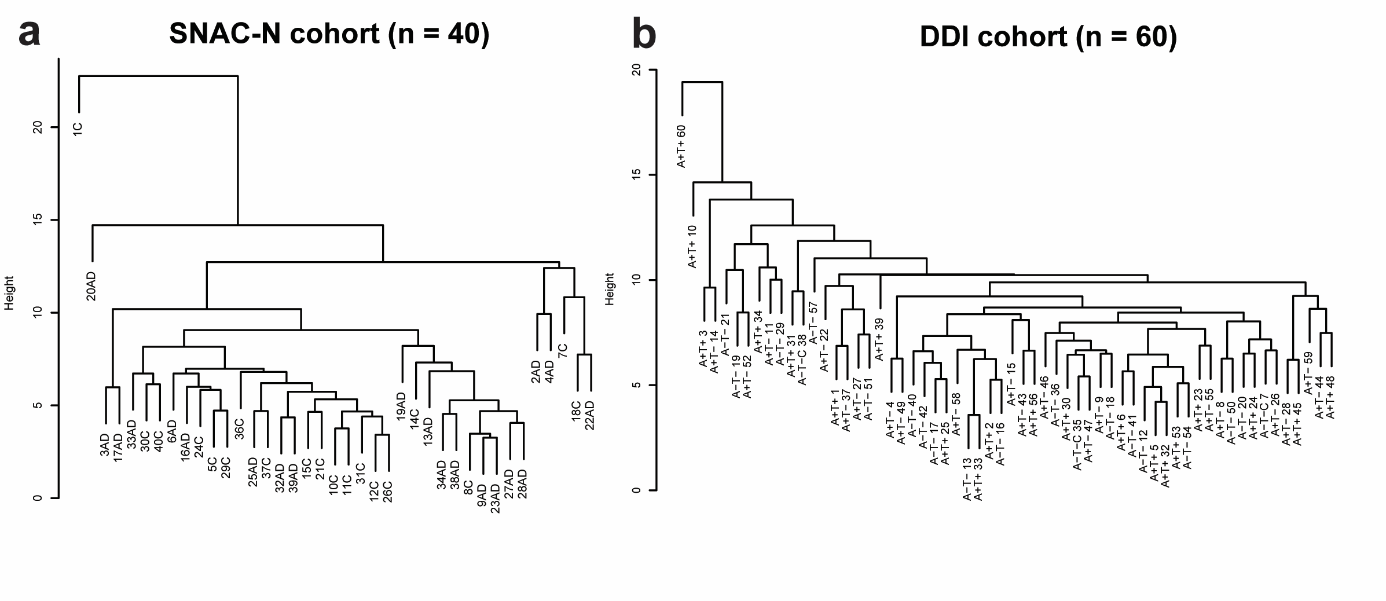


**Figure S2. Identification of outliers with unsupervised hierarchical clustering. (a)** In the Swedish National study on Aging and Care in Nordanstig (SNAC-N) cohort (n = 40), two samples were excluded from further analysis (samples 1 and 20). **(b)** In the Dementia Disease Initiation (DDI) cohort (n = 60), two samples were excluded from further analysis (samples 10 and 60). Hierarchical clustering was performed using the unweighted pair group method with arithmetic mean (UPGMA). Analysis was performed using the hclust function in R.


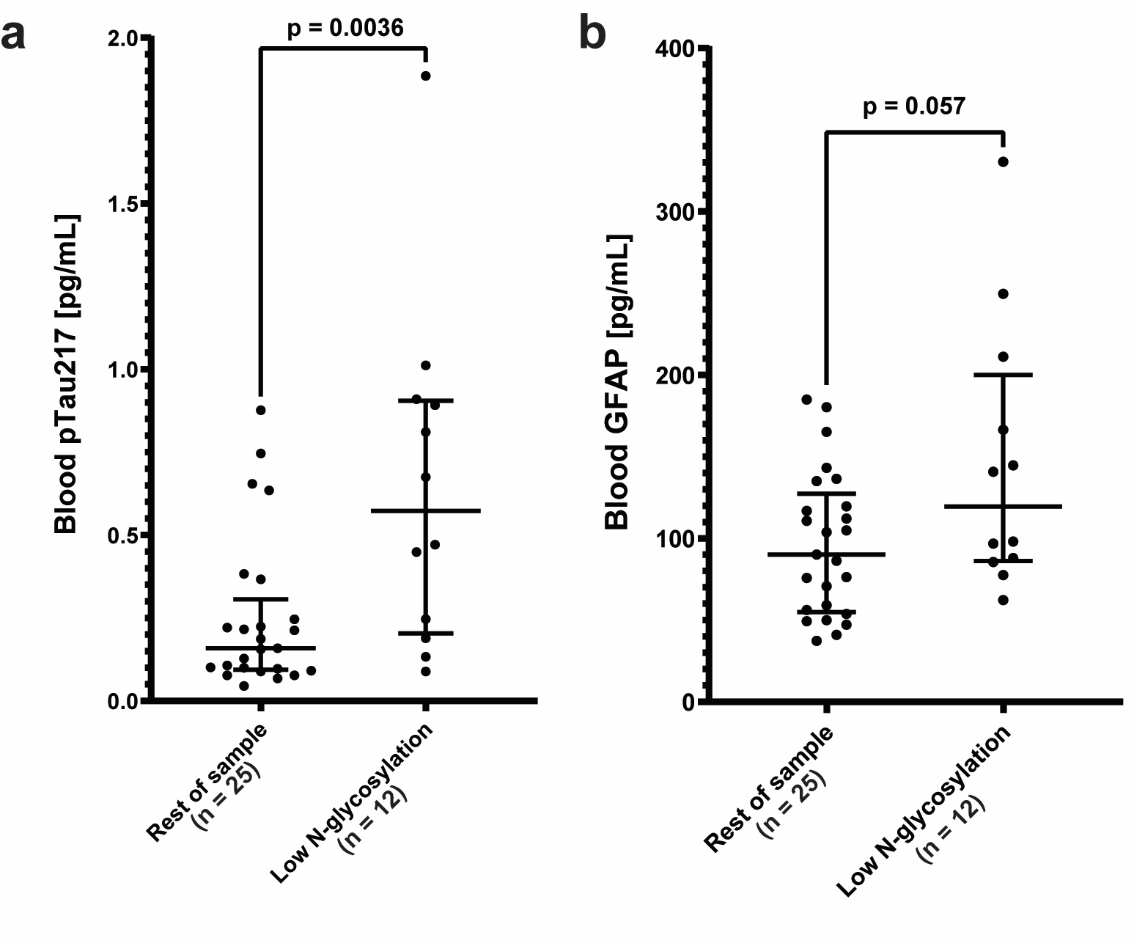


**Figure S3. Blood biomarkers in SNAC-N cohort stratified by plasma N-glycan levels.** (**a**) Individuals with low blood N-glycosylation had higher levels of blood pTau217 (p = 0.0036, Mann-Whitney U-test). **(b)** Levels of blood glial fibrillary acidic protein (GFAP) did not differ significantly between groups (p = 0.057, Mann-Whitney U-test). There was a total of 37 samples. In all graphs, long horizontal lines denote median values, while error bars denote interquartile range.


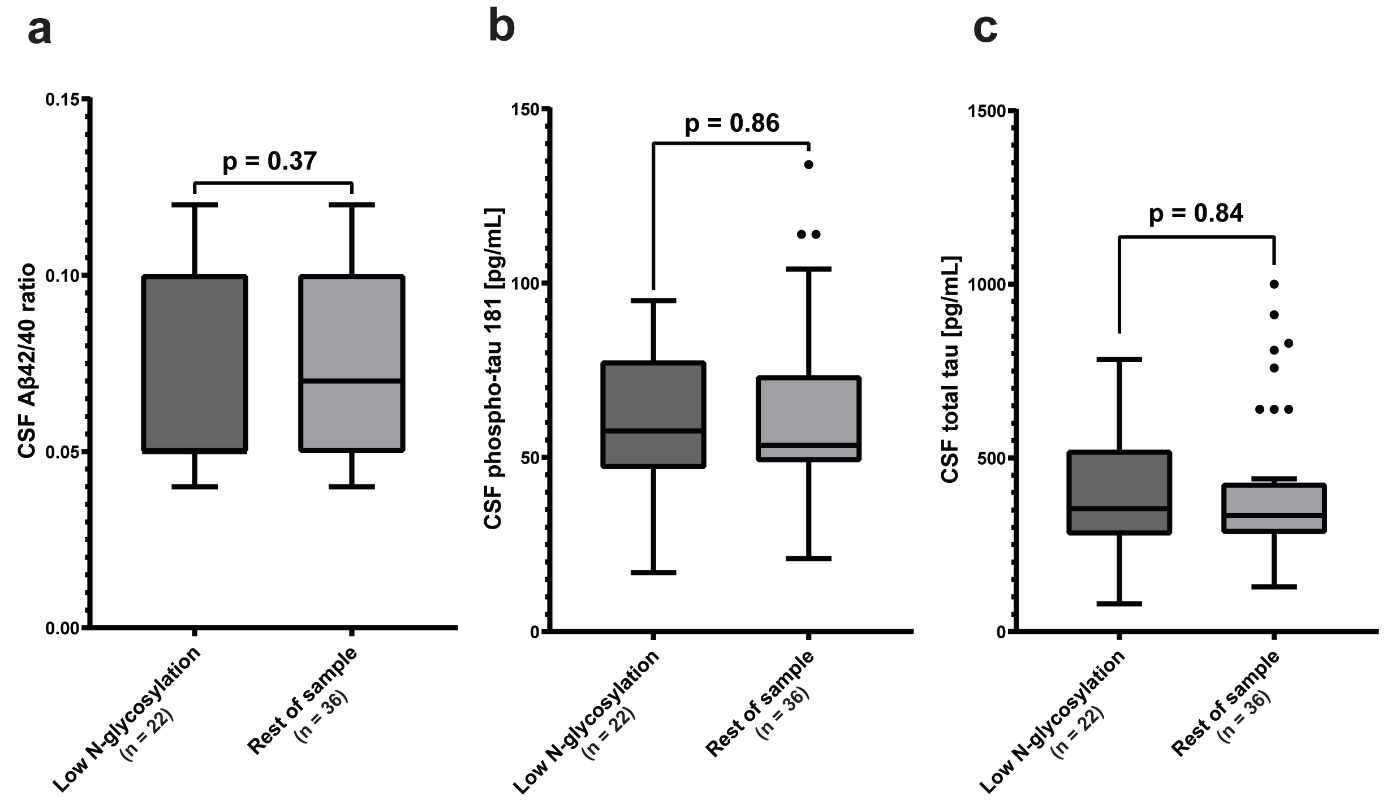


**Figure S4. Cerebrospinal fluid (CSF) biomarker levels in the Dementia Disease Initiation cohort stratified by serum N-glycan levels.** Compared to the rest of the cohort, the low blood N-glycosylation group did not differ in levels of **(a)** CSF amyloid-β peptide 42 to 40 ratio (p = 0.37, Mann-Whitney U-test), **(b)** CSF phospho-tau 181 (p = 0.86, Mann-Whitney U-test), or **(c)** CSF total tau (p = 0.84, Mann-Whitney U-test). There was a total of 58 samples. In all graphs, boxplots were created using the Tukey method, i.e., the solid line indicates the median value, while boxes indicate Q1 and Q3 values. Outliers are indicated by dots and were defined as Q1 - interquartile range (IQR)*1.5 or Q3 + IQR*1.5.


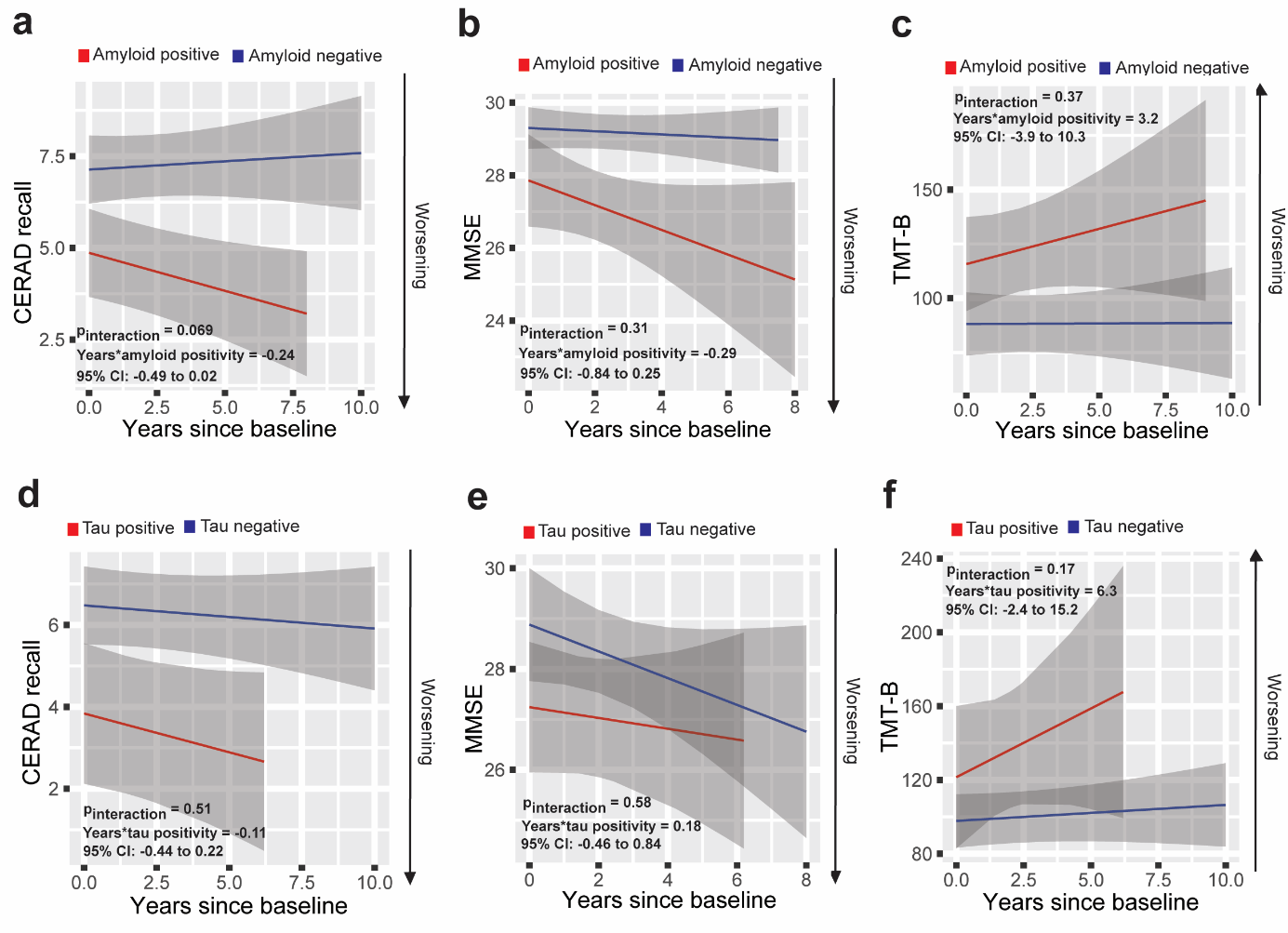


**Figure S5. Longitudinal trajectory of cognitive test scores stratified by baseline amyloid/tau status.** (**a-c**) Trajectory of cognitive test scores in amyloid positive individuals (n = 38) compared to amyloid negative individuals (n = 20). Amyloid positive individuals did not show significantly faster speed of cognitive decline according to CERAD recall score (p = 0.069, Satterthwaite t-test), MMSE score (p = 0.31, Satterthwaite t-test), or TMT-B score (p = 0.37 Satterthwaite t-test). (**d-f**) Trajectory of cognitive test scores in tau positive individuals (n = 18) compared to tau negative individuals (n = 40). Tau positive individuals did not show significantly faster speed of cognitive decline according to CERAD recall score (p = 0.51, Satterthwaite t-test), MMSE score (p = 0.58, Satterthwaite t-test), or TMT-B score (p = 0.17, Satterthwaite t-test).To determine if the rate of cognitive decline was significantly different between groups, we used linear mixed-effects models with cognitive test scores as outcome variables and the interaction term years elapsed*amyloid/tau positivity as predictor variable. Estimates for the interaction terms are presented in the graph, along with 95% confidence intervals and p-values. Graphs were created using the “ggplot2” and “ggeffects” packages in R. Shaded areas indicate 95% confidence bands. Abbreviations: CERAD, Consortium to Establish a Registry for Alzheimer’s Disease; CI, confidence interval; MMSE, Mini-Mental State Examination; TMT-B, Trail Making Test part B.


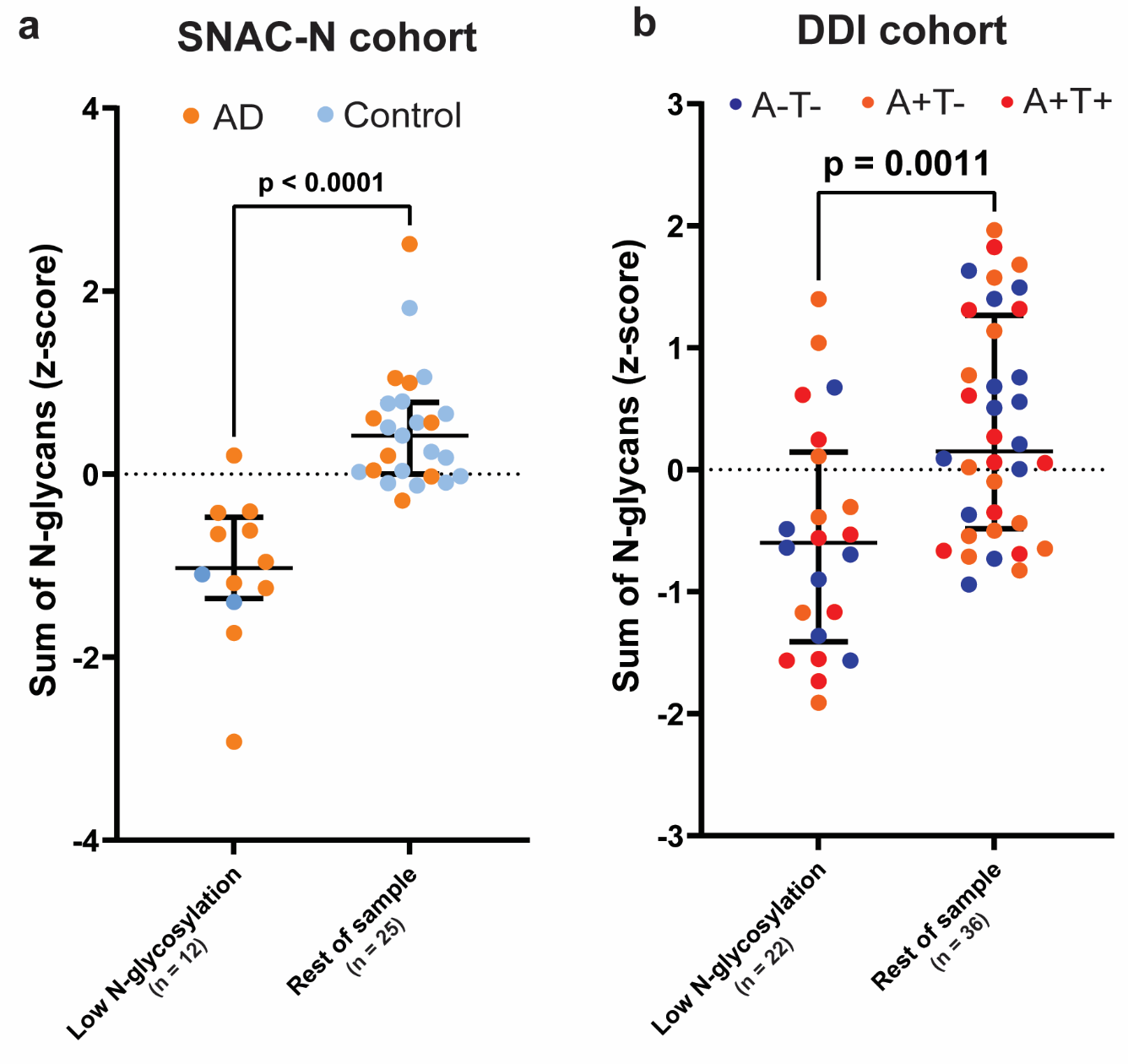


**Figure S6. The total level of blood N-glycans in the low N-glycosylation cluster compared to the rest of the sample.** Each data point represents the Z-standardized sum of all measured N-glycans in one individual in the **(a)** Swedish National study on Aging and Care in Nordanstig (SNAC-N) cohort (n = 37) and the **(b)** Dementia Disease Initiation (DDI) cohort (n = 58). Individuals in the low N-glycosylation group had lower total levels of blood N-glycans compared to the rest of the cohort in both the SNAC-N cohort (p < 0.0001, Mann-Whitney U-test) and the DDI cohort (p = 0.0011, Mann-Whitney U-test). Long horizontal lines denote median values, while error bars denote interquartile range.
