# Supplementary material for: Blood N-glycomics reveals individuals at risk for cognitive decline and Alzheimer’s disease
Source: eBioMedicine. 2025 Feb 20;113:105598. doi: 10.1016/j.ebiom.2025.105598 (PMC11893330; doi:10.1016/j.ebiom.2025.105598)
Supplement: Supplementary Tables [file mmc9.docx]

**Supplement to: Blood N-glycomics reveals individuals at risk for cognitive decline and Alzheimer’s disease.**

Contents

[**Table S1. List of identified N-glycan structures.** 2](#_Toc188475286)

[**Table S2. Linear mixed effects models with longitudinal cerebrospinal fluid biomarkers as outcome variables in the DDI cohort.** 4](#_Toc188475287)

[**Table S3. Linear mixed effects models with longitudinal cognitive test scores as outcome variables in the DDI cohort.** 4](#_Toc188475288)

# **Table S1. List of identified N-glycan structures.**

*contd.*

| **Category** | **Molecule Name** | **Molecule Formula** | **Adduct** | **m/z** | **Charge** | **Retention time (min)** |
| --- | --- | --- | --- | --- | --- | --- |
| Monoantennary | (HexNAc)2 + (Man)3(GlcNAc)2 | C50H86O36N4 | [M-2H] | 658.24 | -2 | 8.92 |
| Internal standard | Stachyose | C24H42O21 | [M-H] | 665.21 | -1 | 8.92 |
| Biantennary | (HexNAc)2 (Deoxyhexose)1 + (Man)3(GlcNAc)2 | C56H96O40N4 | [M-2H] | 731.27 | -2 | 9.83 |
| Biantennary | (Hex)1 (HexNAc)2 + (Man)3(GlcNAc)2 | C56H96O41N4 | [M-2H] | 739.27 | -2 | 9.29 |
| Biantennary | (HexNAc)3 + (Man)3(GlcNAc)2 | C58H99O41N5 | [M-2H] | 759.78 | -2 | 7.75 |
| High mannose | (Hex)4 + (Man)3(GlcNAc)2 #1 | C58H100O46N2 | [M-2H] | 779.27 | -2 | 8.72 |
| High mannose | (Hex)4 + (Man)3(GlcNAc)2 #2-3 | C58H100O46N2 | [M-2H] | 779.27 | -2 | 8.96 |
| Monoantennary | (Hex)1 (HexNAc)1 (NeuAc)1 + (Man)3(GlcNAc)2 | C59H100O44N4 | [M-2H] | 783.28 | -2 | 9.47 |
| Monoantennary | (Hex)1 (HexNAc)2 (Deoxyhexose)1 + (Man)3(GlcNAc)2 | C62H106O45N4 | [M-2H] | 812.30 | -2 | 10.09 |
| Biantennary | (Hex)2 (HexNAc)2 + (Man)3(GlcNAc)2 | C62H106O46N4 | [M-2H] | 820.30 | -2 | 9.62 |
| Triantennary | (HexNAc)3 (Deoxyhexose)1 + (Man)3(GlcNAc)2 | C64H109O45N5 | [M-2H] | 832.81 | -2 | 8.69 |
| Triantennary | (Hex)1 (HexNAc)3 + (Man)3(GlcNAc)2 | C64H109O46N5 | [M-2H] | 840.81 | -2 | 8.17 |
| High mannose | (Hex)5 + (Man)3(GlcNAc)2 | C64H110O51N2 | [M-2H] | 860.30 | -2 | 8.72 |
| Hybrid | (Hex)2 (HexNAc)1 (NeuAc)1 + (Man)3(GlcNAc)2 #1 | C65H110O49N4 | [M-2H] | 864.30 | -2 | 9.21 |
| Hybrid | (Hex)2 (HexNAc)1 (NeuAc)1 + (Man)3(GlcNAc)2 #2 | C65H110O49N4 | [M-2H] | 864.30 | -2 | 10.54 |
| Biantennary | (Hex)1 (HexNAc)2 (NeuAc)1 + (Man)3(GlcNAc)2 #1 | C67H113O49N5 | [M-2H] | 884.82 | -2 | 9.62 |
| Biantennary | (Hex)1 (HexNAc)2 (NeuAc)1 + (Man)3(GlcNAc)2 #2 | C67H113O49N5 | [M-2H] | 884.82 | -2 | 9.88 |
| Biantennary | (Hex)2 (HexNAc)2 (Deoxyhexose)1 + (Man)3(GlcNAc)2 | C68H116O50N4 | [M-2H] | 893.33 | -2 | 10.40 |
| Hybrid | (Hex)1 (HexNAc)3 (deoxyhexose)1 + (Man)3(GlcNAc)2 | C70H119O50N5 | [M-2H] | 913.84 | -2 | 8.99 |
| Triantennary | (Hex)2 (HexNAc)3 + (Man)3(GlcNAc)2 | C70H119O51N5 | [M-2H] | 921.84 | -2 | 8.36 |
| High mannose | (Hex)6 + (Man)3(GlcNAc)2 | C70H120O56N2 | [M-2H] | 941.32 | -2 | 8.80 |
| Hybrid | (Hex)3 (HexNAc)1 (NeuAc)1 + (Man)3(GlcNAc)2 #1 | C71H120O54N4 | [M-2H] | 945.33 | -2 | 9.34 |
| Hybrid | (Hex)3 (HexNAc)1 (NeuAc)1 + (Man)3(GlcNAc)2 #2 | C71H120O54N4 | [M-2H] | 945.33 | -2 | 9.69 |
| Hybrid | (Hex)3 (HexNAc)1 (NeuAc)1 + (Man)3(GlcNAc)2 #3 | C71H120O54N4 | [M-2H] | 945.33 | -2 | 10.85 |
| Biantennary | (Hex)2 (HexNAc)2 (NeuAc)1 + (Man)3(GlcNAc)2 | C73H123O54N5 | [M-2H] | 965.84 | -2 | 9.86 |
| Hybrid | (Hex)1 (HexNAc)3 (NeuAc)1 + (Man)3(GlcNAc)2 | C75H126O54N6 | [M-2H] | 986.36 | -2 | 8.43 |
| Triantennary | (Hex)2 (HexNAc)3 (Deoxyhexose)1 + (Man)3(GlcNAc)2 | C76H129O55N5 | [M-2H] | 994.87 | -2 | 9.21 |
| Biantennary | (Hex)2 (HexNAc)2 (Deoxyhexose)1 (NeuAc)1 + (Man)3(GlcNAc)2 #1 | C79H133O58N5 | [M-2H] | 1038.87 | -2 | 9.63 |
| Biantennary | (Hex)2 (HexNAc)2 (Deoxyhexose)1 (NeuAc)1 + (Man)3(GlcNAc)2 #2 | C79H133O58N5 | [M-2H] | 1038.87 | -2 | 10.58 |
| Biantennary | (Hex)2 (HexNAc)2 (Deoxyhexose)1 (NeuAc)1 + (Man)3(GlcNAc)2 #3 | C79H133O58N5 | [M-2H] | 1038.87 | -2 | 11.66 |
| Hybrid | (Hex)1 (HexNAc)3 (NeuAc)1 (Deoxyhexose) 1+ (Man)3(GlcNAc)2 | C81H136O58N6 | [M-2H] | 1059.39 | -2 | 9.17 |
| Triantennary | (Hex)2 (HexNAc)3 (NeuAc)1 + (Man)3(GlcNAc)2 #1 | C81H136O59N6 | [M-2H] | 1067.38 | -2 | 8.81 |
| Triantennary | (Hex)2 (HexNAc)3 (NeuAc)1 + (Man)3(GlcNAc)2 #2 | C81H136O59N6 | unknown | 1098.42 | -2 | 8.86 |
| Biantennary | (Hex)2 (HexNAc)2 (NeuAc)2 + (Man)3(GlcNAc)2 #1 | C84H140O62N6 | [M-2H] | 1111.39 | -2 | 10.28 |
| Biantennary | (Hex)2 (HexNAc)2 (NeuAc)2 + (Man)3(GlcNAc)2 #2 | C84H140O62N6 | [M-2H] | 1111.39 | -2 | 11.48 |
| Triantennary | (Hex)2 (HexNAc)3 (Deoxyhexose)1 (NeuAc)1 + (Man)3(GlcNAc)2 | C87H146O63N6 | [M-2H] | 1140.41 | -2 | 9.41 |
| Triantennary | (Hex)3 (HexNAc)3 (NeuAc)1 + (Man)3(GlcNAc)2 #1 | C87H146O64N6 | [M-2H] | 1148.41 | -2 | 10.05 |
| Triantennary | (Hex)3 (HexNAc)3 (NeuAc)1 + (Man)3(GlcNAc)2 #2 | C87H146O64N6 | [M-2H] | 1148.41 | -2 | 10.58 |
| Tetraantennary | (Hex)4 (HexNAc)4 (NeuAc)4 + (Man)3(GlcNAc)2 | C134H220O98N10 | [M-3H] | 1178.08 | -3 | 14.54 |
| Biantennary | (Hex)2 (HexNAc)2 (Deoxyhexose)1 (NeuAc)2 + (Man)3(GlcNAc)2 #1-2 | C90H150O66N6 | [M-2H] | 1184.42 | -2 | 10.93 |
| Biantennary | (Hex)2 (HexNAc)2 (Deoxyhexose)1 (NeuAc)2 + (Man)3(GlcNAc)2 #3 | C90H150O66N6 | [M-2H] | 1184.42 | -2 | 12.07 |
| Biantennary | (Hex)2 (HexNAc)2 (Deoxyhexose)1 (NeuAc)2 + (Man)3(GlcNAc)2 #4 | C90H150O66N6 | [M-2H] | 1184.42 | -2 | 13.01 |
| Biantennary | (Hex)2 (HexNAc)3 (NeuAc)2 + (Man)3(GlcNAc)2 | C92H153O67N7 | [M-2H] | 1212.93 | -2 | 9.31 |
| Tetraantennary | (Hex)4 (HexNAc)4 (NeuAc)4 (Deoxyhexose)1 + (Man)3(GlcNAc)2 | C140H230O102N10 | [M-3H] | 1226.76 | -3 | 13.78 |
| High mannose | (Hex)2 + (Man)3(GlcNAc)2 | C46H80O36N2 | [M-H] | 1235.44 | -1 | 10.06 |
| Biantennary | (Hex)2 (HexNAc)2 (Deoxyhexose)2 (NeuAc)2 + (Man)3(GlcNAc)2 | C96H160O70N6 | [M-2H] | 1257.45 | -2 | 11.22 |
| Tetraantennary | (Hex)4 (HexNAc)4 (NeuAc)4 (Deoxyhexose)2 + (Man)3(GlcNAc)2 | C146H240O106N10 | [M-3H] | 1275.45 | -3 | 11.58 |
| Triantennary | (Hex)2 (HexNAc)3 (Deoxyhexose)1 (NeuAc)2 + (Man)3(GlcNAc)2 | C98H163O71N7 | [M-2H] | 1285.96 | -2 | 9.86 |
| Triantennary | (Hex)3 (HexNAc)3 (NeuAc)2 + (Man)3(GlcNAc)2 #1 | C98H163O72N7 | [M-2H] | 1293.96 | -2 | 10.43 |
| Triantennary | (Hex)3 (HexNAc)3 (NeuAc)2 + (Man)3(GlcNAc)2 #2 | C98H163O72N7 | [M-2H] | 1293.96 | -2 | 10.97 |
| Triantennary | (Hex)3 (HexNAc)3 (NeuAc)2 + (Man)3(GlcNAc)2 #3 | C98H163O72N7 | [M-2H] | 1293.96 | -2 | 11.61 |
| Triantennary | (Hex)3 (HexNAc)3 (NeuAc)2 + (Man)3(GlcNAc)2 #4-5 | C98H163O72N7 | [M-2H] | 1293.96 | -2 | 11.87 |
| Triantennary | (Hex)3 (HexNAc)3 (NeuAc)2 (Deoxyhexose)1 + (Man)3(GlcNAc)2 #1-2 | C104H173O76N7 | [M-2H] | 1366.99 | -2 | 10.62 |
| Triantennary | (Hex)3 (HexNAc)3 (NeuAc)2 (Deoxyhexose)1 + (Man)3(GlcNAc)2 #3 | C104H173O76N7 | [M-2H] | 1366.99 | -2 | 11.54 |
| Triantennary | (Hex)3 (HexNAc)3 (NeuAc)2 (Deoxyhexose)1 + (Man)3(GlcNAc)2 #4 | C104H173O76N7 | [M-2H] | 1366.99 | -2 | 12.05 |
| Triantennary | (Hex)3 (HexNAc)3 (NeuAc)2 (Deoxyhexose)1 + (Man)3(GlcNAc)2 #5-7 | C104H173O76N7 | [M-2H] | 1366.99 | -2 | 13.09 |
| High mannose | (Hex)3 + (Man)3(GlcNAc)2 | C52H90O41N2 | [M-H] | 1397.49 | -1 | 9.00 |
| Triantennary | (NeuAc)3 (Hex)3 (HexNAc)3 + (Man)3(GlcNAc)2 #1 | C109H180O80N8 | [M-2H] | 1439.51 | -2 | 11.73 |
| Triantennary | (NeuAc)3 (Hex)3 (HexNAc)3 + (Man)3(GlcNAc)2 #2 | C109H180O80N8 | [M-2H] | 1439.51 | -2 | 12.22 |
| Triantennary | (NeuAc)3 (Hex)3 (HexNAc)3 + (Man)3(GlcNAc)2 #3 | C109H180O80N8 | [M-2H] | 1439.51 | -2 | 13.19 |
| Triantennary | (Hex)3 (HexNAc)3 (NeuAc)3 (Deoxyhexose)1 + (Man)3(GlcNAc)2 #1 | C115H190O84N8 | [M-2H] | 1512.53 | -2 | 11.38 |
| Triantennary | (Hex)3 (HexNAc)3 (NeuAc)3 (Deoxyhexose)1 + (Man)3(GlcNAc)2 #2-3 | C115H190O84N8 | [M-2H] | 1512.53 | -2 | 12.61 |
| Tetraantennary | (Hex)4 (HexNAc)4 (NeuAc)3 + (Man)3(GlcNAc)2 | C123H203O90N9 | [M-2H] | 1622.07 | -2 | 13.34 |

Abbreviations: GlcNAc, N-acetylglucosamine; Hex, hexose; HexNAc, N-acetylhexosamine; Man, mannose; m/z, mass to charge ratio; NeuAc, neuraminic acid

**Table S2. Linear mixed effects models with longitudinal cerebrospinal fluid biomarkers as outcome variables in the DDI cohort.**

| **Dependent variable** | **Independent variable** | **Estimate** | **95% CI** | **df** | **p-value** |
| --- | --- | --- | --- | --- | --- |
| **Aβ42/40** |  |  |  |  |  |
|  | Intercept | 0.0743 | 0.0652 to 0.0834 | 47.222 | <0.0001 |
|  | Years | -0.00298 | -0.00430 to -0.00163 | 43.591 | <0.0001 |
|  | Low glycosylation | -0.0105 | -0.0256 to 0.00462 | 47.145 | 0.18 |
|  | Years*Low N-glycosylation | 0.00104 | -0.00118 to 0.00322 | 44.014 | 0.36 |
| **pTau181** |  |  |  |  |  |
|  | Intercept | 61.781 | 54.529 to 69.047 | 52.614 | <0.0001 |
|  | Years | 2.077 | -1.009 to 5.048 | 34.026 | 0.19 |
|  | Low glycosylation | 4.470 | -7.563 to 16.516 | 52.090 | 0.47 |
|  | Years*Low N-glycosylation | 4.223 | -0.222 to 8.954 | 33.521 | 0.075 |
| **Total tau** |  |  |  |  |  |
|  | Intercept | 400.426 | 338.450 to 462.518 | 47.376 | <0.0001 |
|  | Years | 19.575 | 0.247 to 38.118 | 27.647 | 0.051 |
|  | Low glycosylation | 43.478 | -59.534 to 146.515 | 47.126 | 0.41 |
|  | Years*Low N-glycosylation | 26.398 | -1.250 to 55.615 | 27.444 | 0.076 |

The interaction term of low blood N-glycosylation*years elapsed was tested. Abbreviations: Aβ42/40, amyloid-β peptide 42 to 40 ratio; CI, confidence interval; df, degrees of freedom; pTau181, phosphorylated tau-181

| **Dependent variable** | **Independent variable** | **Estimate** | **95% CI** | **df** | **p-value** |
| --- | --- | --- | --- | --- | --- |
| **CERAD recall** |  |  |  |  |  |
|  | Intercept | 5.943 | 4.863 to 7.022 | 48.480 | <0.0001 |
|  | Years | 0.0717 | -0.083 to 0.232 | 69.262 | 0.37 |
|  | Low glycosylation | -0.913 | -2.705 to 0.879 | 48.187 | 0.32 |
|  | Years*Low N-glycosylation | -0.397 | -0.652 to -0.143 | 69.950 | 0.0032 |
| **MMSE** |  |  |  |  |  |
|  | Intercept | 27.989 | 26.948 to 29.026 | 109.851 | <0.0001 |
|  | Years | 0.146 | -0.209 to 0.510 | 107.124 | 0.43 |
|  | Low glycosylation | 0.750 | -0.938 to 2.453 | 107.020 | 0.39 |
|  | Years*Low N-glycosylation | -0.761 | -1.318 to -0.218 | 114.715 | 0.0065 |
| **TMT-B** |  |  |  |  |  |
|  | Intercept | 109.428 | 90.828 to 127.994 | 65.032 | <0.0001 |
|  | Years | -1.541 | -6.015 to 2.878 | 80.857 | 0.50 |
|  | Low glycosylation | -8.482 | -39.478 to 22.445 | 63.436 | 0.60 |
|  | Years*Low N-glycosylation | 7.882 | 0.824 to 14.903 | 84.926 | 0.031 |

**Table S3. Linear mixed effects models with longitudinal cognitive test scores as outcome variables in the DDI cohort.**

The interaction term of low blood N-glycosylation*years elapsed was tested. Abbreviations: CERAD, Consortium to Establish a Registry for Alzheimer’s disease; CI, confidence interval; df, degrees of freedom; MMSE. Mini-Mental State Examination; TMT-B, Trail-Making Test part B
